# Surfactant Protein D Reverses the Gene Signature of Transepithelial HIV-1 Passage and Restricts the Viral Transfer Across the Vaginal Barrier

**DOI:** 10.3389/fimmu.2019.00264

**Published:** 2019-03-28

**Authors:** Hrishikesh Pandit, Kavita Kale, Hidemi Yamamoto, Gargi Thakur, Sushama Rokade, Payal Chakraborty, Madavan Vasudevan, Uday Kishore, Taruna Madan, Raina Nakova Fichorova

**Affiliations:** ^1^Department of Innate Immunity, ICMR National Institute for Research in Reproductive Health, Mumbai, India; ^2^Laboratory of Genital Tract Biology, Harvard Medical School and Brigham and Women's Hospital, Boston, MA, United States; ^3^Genome Informatics Research Group, Bionivid Technology Pvt. Ltd., Bengaluru, India; ^4^Biosciences, College of Health and Life Sciences, Brunel University London, Uxbridge, United Kingdom

**Keywords:** surfactant protein D, HIV-1, vaginal, microarray, chemokines, microbicide

## Abstract

Effective prophylactic strategy against the current epidemic of sexually transmitted HIV-1 infection requires understanding of the innate gatekeeping mechanisms at the genital mucosa. Surfactant protein D (SP-D), a member of the collectin family of proteins naturally present in the vaginal tract, is a potential HIV-1 entry inhibitor at the cellular level. Human EpiVaginal tissues compartmentalized in culture inserts were apically exposed to HIV-1 and/or a recombinant fragment of human SP-D (rfhSP-D) and viral passage was assessed in the basal chamber containing mononuclear leukocytes. To map the gene signature facilitating or resisting the transepithelial viral transfer, microarray analysis of the HIV-1 challenged EpiVaginal tissues was performed in the absence or presence of rfhSP-D. Mucosal biocompatibility of rfhSP-D was assessed *ex vivo* and in the standard rabbit vaginal irritation model. The passage of virus through the EpiVaginal tissues toward the underlying target cells was associated with a global epithelial gene signature including differential regulation of genes primarily involved in inflammation, tight junctions and cytoskeletal framework. RfhSP-D significantly inhibited HIV-1 transfer across the vaginal tissues and was associated with a significant reversal of virus induced epithelial gene signature. Pro-inflammatory NF-κB and mTOR transcripts were significantly downregulated, while expression of the tight junctions and cytoskeletal genes was upheld. In the absence of virus, rfhSP-D directly interacted with the EpiVaginal tissues and upregulated expression of genes related to structural stability of the cell and epithelial integrity. There was no increment in the viral acquisition by the PBMCs present in basal chambers wherein, the EpiVaginal tissues in apical chambers were treated with rfhSP-D. The effective concentrations of rfhSP-D had no effect on *lactobacilli*, epithelial barrier integrity and were safe on repeated applications onto the rabbit vaginal mucosa. This pre-clinical safety data, coupled with its efficacy of restricting viral passage via reversal of virus-induced gene expression of the vaginal barrier, make a strong argument for clinical trials of rfhSP-D as a topical anti-HIV microbicide.

## Introduction

A clear majority of the HIV-1 infections are due to heterosexual contact; more than 50% of HIV-1 infected individuals are women and most children living with HIV-1 today are infected via mother-to-child transmission (1). Thus, an effective vaginal microbicide for the prevention of sexual transmission of HIV-1 to women will have a huge impact on limiting the HIV epidemic and its devastating consequences for both adults and children. Despite this well-perceived need of intervention and the efforts made to date in understanding the vaginal mucosal barrier (2–4), the development of a safe and effective topical vaginal microbicide has several technical challenges (5–7). Clinical trials involving most of the promising candidates showed reduced efficacy as they adversely affected the vaginal milieu (7). Evaluation of microbicides *in vivo* using SIV-macaque and humanized mouse models comes at a high cost and the findings may only be an extrapolation to HIV-1 transmission in humans (7). A serious limitation is lack of an appropriate *ex vivo* model for the evaluation of efficacy of potential compounds on the viral passage across the vaginal barrier to the target immune cells (8–11). The *ex vivo* model should also assess compatibility of the candidate molecules with the mucosal integrity and barrier function including the colonization with healthy vaginal microbiome.

Of special interest for pharmaceutical development are candidate microbicides that would regulate vaginal innate immune responses with minimal adverse effects on the physiology (12, 13). Collectins are a group of secreted, anti-microbial pattern recognition proteins in the female reproductive tract (14–17). Surfactant Protein D (SP-D) is one such collectin expressed by the epithelium, lining the vaginal tract (18). Previously, we have demonstrated that a recombinant fragment of human SP-D (rfhSP-D) containing homotrimeric neck and C-type lectin domains binds to HIV-1 envelope glycoprotein gp120, and inhibits viral entry and replication in target immune cells (19). Beyond its pattern recognition capability, SP-D interacts with various immune cells, maintains Th1/Th2 balance in the lungs and induces immune quiescence (20, 21). By virtue of its natural presence in the vaginal tract, broad anti-microbial activity and immune-regulatory functions, SP-D is a unique microbicide candidate. Importantly, anti-HIV-1 activity of rfhSP-D was intact in physiological fluids like vaginal lavage and seminal plasma which comprise of diverse enzymes, pH and inhibitors (19).

In this study, we assessed the effect of rfhSP-D on the interactions of vaginal epithelial tissues and HIV-1 using a rational scheme for *ex vivo* microbicide testing. The scheme is designed to resemble sexual transmission of the virus and comprises of bioengineered vaginal tissues, immune cells and clinical isolates of *Lactobacillus*. In our model, HIV-1 traverses through the intact, multi-layered vaginal epithelium toward the underlying mononuclear leukocytes. We report, for the first time, a “gatekeeping” gene signature of bioengineered human tissues induced upon HIV-1 exposure. In this model, rfhSP-D showed no adverse effects on the vaginal barrier, concomitant with a significant impediment of viral movement to the activated PBMCs in the basal compartment. Epithelial transcriptome revealed reversal of HIV-1 induced differential expression of genes associated with the cytoskeleton, inflammation and barrier integrity. A range of preclinical assays confirmed safety of rfhSP-D for vaginal application at the similar concentrations it restricted viral transfer *ex vivo*, and thus, establishing it as a promising anti-HIV-1 vaginal microbicide.

## Materials and Methods

### Human Cell Lines

Well-characterized and immortalized human vaginal (Vk2/E6E7, ATCC® CRL-2616™), endocervical (End1/E6E7, ATCC® CRL-2615™), and ectocervical (Ect1/E6E7, ATCC® CRL-2614™) cell lines developed by Dr. Raina Fichorova (22), were cultured in antibiotic-free keratinocyte serum-free medium (KSFM), supplemented with 50 μg/ml bovine pituitary extract, 0.1 ng/ml epidermal growth factor (Gibco, Invitrogen, USA), and 0.4 mM CaCl_2_ (Fisher Scientific, USA). These cell lines are known to retain their physiological characteristics and are useful models for various female reproductive tract infections, including HIV-1 (22–26).

### Vaginal Bioengineered Tissue (EpiVaginal Tissue)

Twenty four EpiVaginal™ (VEC-100™) tissues and medium were purchased from MatTek (Ashland, MA, USA). These tissues are derived from primary human ectocervical/vaginal epithelial cells, and possess characteristics comparable to that of the normal tissues of origin (26, 27).

### Clinical *Lactobacillus* Isolates

*Lactobacillus crispatus* isolates were obtained from vaginal swab samples of healthy women participating in a vaginal microflora research study at the Brigham and Women's Hospital (Boston, MA, USA) (6). *Lactobacillus fermentum spps mucosae* (TRF#36), *Lactobacillus gasseri* (TRF#8), and *Lactobacillus salivarius* (TRF#30) were a kind gift from Prof. GP Talwar, the Talwar Research Foundation (New Delhi, India) (28).

### Preparation of rfhSP-D

A recombinant fragment of human SP-D (rfhSP-D), composed of trimeric neck and lectin domains along with 8 Gly-X-Y repeats, was expressed in *E. coli*, purified and characterized, as described previously (19, 29, 30). The endotoxin level in the rfhSP-D preparations was determined using the QCL-1000 *Limulus amebocyte* lysate system (BioWhittaker Inc., USA). The endotoxin concentration in the various preparations ranged between 2.8 and 5.1 pg/μg of rfhSP-D. Controls of various experiments were spiked by adding equivalent amounts of LPS (Sigma-Aldrich, USA).

### Assessment of the Expression of SP-D in Human Vaginal Cells (VK2/E6E7) and Cervicovaginal Lavage (CVL)

To assess the presence of SP-D in CVL, total protein was precipitated using chilled acetone; 25 μg total protein was loaded per well and subjected to 12% SDS-PAGE under reducing conditions and then electrophoretically transferred to a nitrocellulose membrane for immuno-blotting. Mouse monoclonal anti-human SP-D antibody (Abcam, UK) was used at a dilution of 1:500, whereas, rabbit polyclonal anti-mouse secondary antibody conjugated to horseradish peroxidase (HRP) was used at a dilution of 1:1,000 (Dako). Detection was done using chemiluminescent detection kit (Amersham Biosciences, Piscataway, NJ). For immunostaining, Vk2/E6E7 cells were grown on cover slips, probed with the mouse monoclonal anti-human SP-D antibody (Abcam) and further detected with anti-mouse Phycoerythrin-conjugates (Molecular Probes). Nuclei were counterstained with 4′,6-Diamidine-2′-phenylindole dihydrochloride (DAPI) (Sigma-Aldrich), the coverslips were mounted in Vectashield (Vector Laboratories) and visualized under a confocal microscope (Zeiss, Germany). To determine transcript levels of SP-D, total RNA was extracted using Trizol (Invitrogen) from Vk2/E6E7 cells; 3 μg of total RNA was reverse transcribed into cDNA using Superscript III first strand synthesis kit (Invitrogen) and subjected to PCR (Veriti Machine, Applied Biosystems). Primers for SP-D, SP-A2, and 18S were designed using NCBI Primer BLAST Software (Supplementary Table 1). The resultant PCR products were electrophoresed on a 2% agarose gel at 100V on electrophoresis. The bands were detected via ethidium bromide under UV light.

### *Ex vivo* Model of Vaginal HIV-1 Transmission

**Figure 1:**
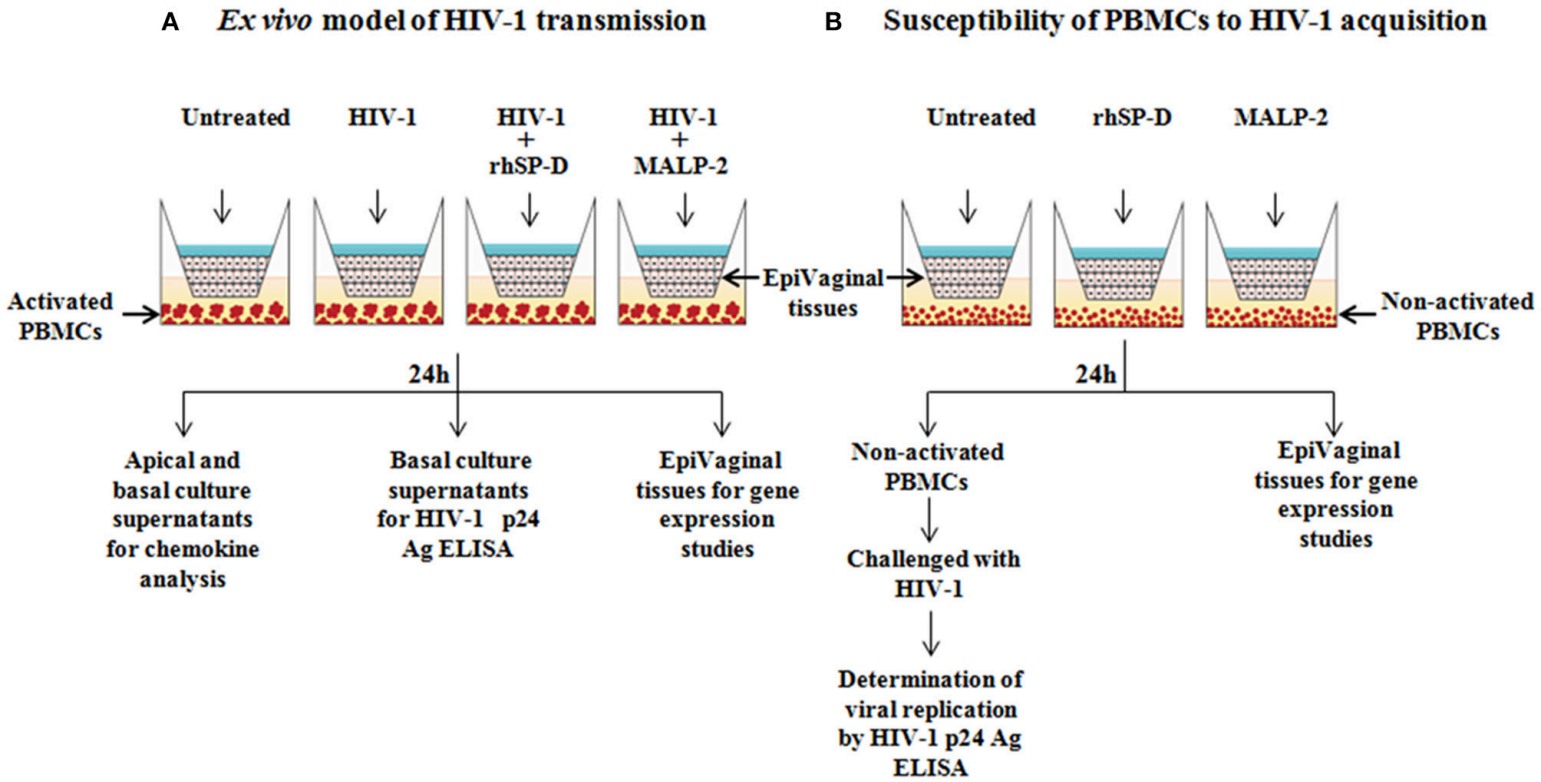
Experimental designs. **(A)** *Ex vivo* model for vaginal transmission of HIV-1. Reconstructed, multi-layered EpiVaginal tissues placed in the cell culture inserts were challenged with HIV-1 in the apical chamber for 24 h. The activated PBMCs placed in the basal chamber served as HIV-1 targets when HIV-1 traversed through the EpiVaginal tissues. Twenty minutes prior to the HIV-1 challenge, the EpiVaginal tissues were either treated with rfhSP-D (HIV-1 entry inhibitor) or MALP-2 (inflammatory TLR agonist). **(B)** *Ex vivo* model to predict susceptibility of basal PBMCs to HIV-1 acquisition. Reconstructed, multi-layered EpiVaginal tissues placed in the cell culture inserts were either treated with rfhSP-D (HIV-1 entry inhibitor) or MALP-2 (inflammatory TLR agonist). The non-activated PBMCs were placed in the basal chamber to determine if the rfhSP-D and MALP-2 treatment of the vaginal tissue may lead to secretion of certain mediators resulting in the activation of PBMCs. These treated PBMCs from the basal chamber were subsequently challenged with HIV-1 to evaluate their susceptibility to virus acquisition.

In order to mimic vaginal transmission of HIV-1, a novel *ex vivo* model was developed using EpiVaginal tissues (Figure 1A). Upon delivery, EpiVaginal tissues were acclimatized in the medium overnight. Blood (from non-autologous donors) was subjected to Ficoll separation and PBMCs were isolated. PBMCs were activated for 48 h using rhIL-2 (100 U/ml) (Sigma-Aldrich) and PHA (5 μg/ml) in the RPMI 1640 medium (Fisher Scientific) containing 10% FBS and 0.5% antibiotic solution (Gibco, Invitrogen). Activated PBMCs (10^5^) were seeded in a 12-well plate as target cells for further replication of migrated virions. In a fresh tissue culture plate, inserts containing EpiVaginal tissues were placed in each well. In our previous study, we have reported the anti-HIV activity of purified native human SP-D, rfhSP-D and another variant of recombinant fragment of SP-D, lacking eight triplets of collagen repeats (delta-rfhSP-D) (19). Although delta-rfhSP-D showed anti-HIV activity in the TZMbl reporter assays (IC_50_ 43.282 ± 10.76 μg/ml), it was 3-fold less potent than the native human SP-D and rfhSP-D (IC_50_ of rfhSP-D with various viral isolates and target cell types ranged between 6.726 ± 0.63 and 13.676 ± 3.37 μg/ml) (19). In another assay (as described in the section “Viability MTT assay”), we evaluated the effect of various concentrations of rfhSP-D (1.562–100 μg/ml) on the viability of vaginal epithelial cells and used the maximal tolerated dose of rfhSP-D (100 μg/ml) in the *ex vivo* model. The physiological concentration of free SP-D in various body fluids ranges from 0.5 to ~3 μg/ml (31, 32). In view of the ability of SP-D to bind several molecules, such as immunoglobulins, fatty acids and nucleic acids, its total physiological concentration is expected to be much higher (33–35). Apical tissues were treated with rfhSP-D (100 μg/ml) or a synthetic analog of *Mycoplasma fermentans* lipopeptide macrophage activating lipopeptide 2 (MALP-2) (Alexis Biochemicals, USA) (25 nM) for 20 min before inoculation with 100 TCID_50_ R5 tropic HIV-1_JR−CSF_. After 24 h incubation, apical and basal supernatants were collected for determining levels of immune mediators. Basal supernatants were used to determine HIV-1 p24 Ag by ELISA, as per manufacturer's instructions (R&D Systems).

### Microarray Gene Expression Analysis

The microarray data, described in this study, has been deposited in the NCBI Gene Expression Omnibus (GEO) under the GEO series accession number GSE107478.

#### RNA Isolation

Total RNA was extracted using TRIZOLVR Reagent (Invitrogen); RNA quantity and quality were determined using NanodropVR spectrophotometer (NanoDrop Technologies, Wilmington, DE). Targets were prepared using the Illumina RNA amplification kit (Ambion, Austin, TX). cRNA was synthesized from 200 ng of the total RNA followed by amplification and labeling steps. Amplified biotin-labeled cRNA was hybridized to the Illumina Human HT12 V6 bead chip. Illumina Bead Studio was used to extract the raw data from the bead chip. Raw data was Quantile normalized and baseline transformation was carried out to obtain median of all samples using GeneSpring GX 12.5 software (Agilent Technologies Inc, Santa Clara, USA).

#### Statistical Analysis and Differentially Expressed Genes

Differentially expressed probe sets (genes) in the treated cells in comparison to the untreated cells were identified by applying Volcano Plot using a fold-change threshold (absolute fold-change >1.5). A statistically significant “*t*-test” “*P*-value” threshold was adjusted for false discovery rate of <0.001. Statistically significantly enriched transcripts with a “*P*-value” adjusted for false discovery rate of <0.05, based on the hyper-geometric distribution test corresponding to differentially expressed genes, were determined using the Student's “*t*-test” along with Benjamini Hocheberg FDR test. Unsupervised hierarchical clustering of the differentially expressed genes following treatment in comparison to the untreated cells was performed using Euclidian algorithm with Centroid linkage rule to identify gene clusters whose expression levels were significantly reproduced across the replicates.

#### Biological Pathways and Gene Ontology Enrichment Analysis

Differentially expressed gene list was subjected to a biological significance analysis by GOElite tool. A total of 21,887 protein coding genes were used as the background and the differentially expressed gene list was used as query. Database of GeneOntology categories, Wikipathways, KEGG Pathways, Pathway Commons, Pheno Ontology, Diseases, Protein Domains, Transcription factor targets, and tissue expression were configured for significance analysis. Each query list was subjected to the “Over-representation Analysis” against each of the above databases. Z score and permutation or Fisher's Exact Test *p*-value were calculated to assess over-representation of the enriched biological categories.

#### Biological Analysis Network Modeling of Differential Regulome

Enriched biological categories, along with the differentially expressed genes, were used as input for BridgeIsland Software (Bionivid, Bangalore, India) for identifying the key edges that connect genes with biological categories. Statistical scores from differential expression and biological analysis were used as attributes to visualize the network. Output of BridgeIsland Software was used as input to CytoScape V 2.8. Circular layout and yFiles algorithm were used to visualize the network that encompasses biological categories. Further to this core network, all the differentially expressed genes were colored based on their fold change to reflect the rfhSP-D treatment induced differential regulome.

#### Validation by Real Time RT-PCR

Since, EpiVaginal tissues (of ectocervical origin) used in the assays were sufficient enough for microarray analysis, we carried out the validation of microarray data using an ectocervical cell line (Ect1/E6E7) under conditions similar to the *ex vivo* model of HIV-1 transmission (same MOI). Cells were seeded in a 96-well plate, grown up to confluence and then treated with rfhSP-D (100 μg/ml) for 20 min, before inoculation with 100 TCID_50_ R5 tropic HIV-1_JR−CSF_ at 37°C for 24 h. Total RNA was isolated using Trizol (Invitrogen) and the quality of RNA was assessed by nano-spectrophotometry and the nucleotide: protein ratio (260:280) was determined. 1–3 μg of RNA was reverse transcribed into cDNA using Superscript III first strand synthesis kit (Invitrogen). The resulting cDNA was used for real time PCR via the Bio-Rad CFX96 TouchTM real-time PCR detection system using the iQTM SYBR Green Supermix (Bio-Rad, USA). 18s RNA was used as the housekeeping control. Primers were designed using NCBI Primer BLAST Software. Primer sequences and conditions are provided in the Supplementary Table 1.

### mRNA Levels of Tight Junction Proteins in EpiVaginal Tissues After HIV-1 Challenge

In order to determine the status of the vaginal barrier after the viral challenge, transcripts from EpiVaginal tissues for the tight junction proteins viz. Claudin 2, 3, 4, 5, and occludin were quantified using real time qPCR. Owing to the limited EpiVaginal tissue, the qPCR analysis was not extended to quantitation of protein levels. Primers sequences were synthesized (Sigma-Aldrich) as reported previously (36). Primer sequences and conditions are provided in the Supplementary Table 1.

### Susceptibility of PBMCs to HIV-1 Acquisition

HIV-1 is known to replicate faster in the activated human PBMCs (37). We have shown previously that rfhSP-D rfhSP-D does not alter the activation alter the activation of PBMCs and leads to induction of quiescence in the activated PBMCs (21). The present assay was designed to specifically determine the impact of supernatants from rfhSP-D treated EpiVaginal tissues on the activation status and viral acquisition of PBMCs. Non-activated PBMCs (10^5^) were seeded in a 12-well plate and apical regions of the culture inserts containing EpiVaginal tissues were treated with rfhSP-D (100 μg/ml), MALP-2 (25 nM), or left untreated for 24 h. Following incubation, basal PBMCs were collected and challenged with 100 TCID_50_ HIV-1_JR−CSF_ for 4 h to assess the rate of HIV-1 acquisition (Figure 1B). PBMCs were washed and cultured further in RPMI 1640 medium containing 10% FBS and 1% antibiotic solution for 7 days, and HIV-1 p24 levels in culture supernatants were measured. Viability of PBMCs was evaluated at the end of the assay (data not shown).

### MTT Viability Assay

To assess the likely effect of rfhSP-D on cell viability, MTT assay was performed on Vk2/E6E7 and Ect1/E6E7 cell monolayers. Cells were seeded in a 96-well plate, grown up to confluence and then treated with a range of rfhSP-D concentrations at 37°C for 24 h. Culture supernatants were then collected for measuring immune mediators. Cells were treated with 1 × MTT containing KSFM and incubated overnight; 0.04 N acidified isopropanol was added to the cells to dissolve the formazan crystals. This color intensity, read at OD_570_, is directly proportional to the number of viable cells, as measured by a Victor2 counter with Wallac 2.01 software (PerkinElmer Life Sciences, USA) using a reference wavelength at 630 nm. The OD of untreated (medium alone) control cells was considered as 100%; percent viability of rfhSP-D treated cells was calculated as compared to untreated control.

### NF-κB Luciferase Assay

End1/E6E7 immortalized epithelial cells were transfected with pHTS–NF-κB firefly luciferase reporter vector (Biomyx Technology, USA) using a gene-juice transfection protocol (38). Cells were seeded in a 96-well plate, grown until confluent monolayers and treated with indicated concentrations of rfhSP-D for 24 h at 37°C. A synthetic analog of viral double-stranded RNA, Poly (I:C) (10 μg/ml) (InvivoGen, USA), a TLR3 agonist, and MALP-2 (25 nM), a TLR2/6 agonist, were used as positive controls. After incubation, the supernatant was removed, cells were lysed in GloLysis buffer, and activation of luciferase was determined using a Bright-Glo luciferase assay system (Promega, USA). Luminescence signal was quantified via a Victor2 1420 multi-label microplate counter with Wallac 2.01 software (PerkinElmer Life Sciences).

### Assay for Toxicity to *Lactobacillus*

Direct toxicity assay on vaginal lactobacilli was performed using a colorimetric assay as described previously (39). TRF#8, TRF#30, TRF#36, and *Lactobacillus crispatus* LC223 were grown in the *Lactobacillus* MRS Broth (HiMedia™ Laboratories). Bacterial density was adjusted to an OD_670_ of 0.06, corresponding to a 0.5 McFarlands turbidity standard or ca. 10^8^ CFU/ml. RfhSP-D was plated at the appropriate concentrations into a 96-well round bottom plates in a volume of 100 μl, and the diluted *Lactobacillus spps* were added in a volume of 100 μl. Commercially available penicillin-streptomycin solution (Gibco, Invitrogen) at a maximal test concentration of 1.25 U/ml and 1.25 μg/ml respectively) was used as a positive control for toxicity. Plates were incubated in an orbital shaker at 35°C under anaerobic conditions using AnaeroPack system (PML Microbiologicals, Wilsonville, OR) for 24 h. Bacterial growth was determined by measurement of the OD_490_ using a Victor2 counter with Wallac 2.01 software (PerkinElmer Life Sciences) (40).

### *Lactobacilli*-Epithelial Colonization Assay

Colonization of epithelial cells by lactobacilli in presence of rfhSP-D was assayed as described earlier (6). Briefly, the *Lactobacillus crispatus* isolate, suspended in antibiotic-free KSFM (2.2 × 10^6^ CFU/cm^2^), was added to confluent epithelial surfaces Vk2/E6E7 and End1/NF-κB cells (10:1 ratio) and allowed to adhere on the epithelial monolayer; unbound bacteria were washed off by two washes of sterile Dulbecco's phosphate-buffered saline (PBS) (Invitrogen). To the bacteria-epithelial cell co-culture, indicated concentrations of rfhSP-D, Poly (I:C) or MALP-2 was added to each well. Supernatants were collected after 24 h to measure immune mediators. Vk2/E6E7 epithelial cells were washed twice with sterile PBS and examined for viability by MTT (data not shown) and colony forming unit (CFU) assays. End1/NF-κB co-culture plate was used to evaluate NF-κB activation.

### Colony Forming Units (CFU) Counts

Viable bacteria associated with Vk2/E6E7 monolayers were measured by CFU counts after 24 h of epithelial colonization followed by 24 h exposure to rfhSP-D (6). To enumerate cell-associated bacteria, epithelial cells were washed with cold PBS and hypotonically lysed in ice-cold HyPure water for 15 min, followed by adjustment of osmolality with PBS (2X) (Fisher Scientific). Bacteria collected were plated on Brucella anaerobic agar with 5% sheep blood (Becton, Dickinson and Company, USA) as per the standardized protocol, and incubated in an anaerobic chamber (Coy Laboratory Products, USA) (10% hydrogen, 10% carbon dioxide, and 80% nitrogen) at 35°C for up to 72 h (until colonies were formed), followed by visual counting of CFU.

### Quantitation of Immune Mediators

Culture supernatants from the EpiVaginal tissues (apical), PBMCs (basal) and Vk2/E6E7 (with or without bacterial co-culture) were collected separately from various experiments. A custom designed 3-plex assay for GRO-α (CXCL1), MIP-3α (CCL20), and RANTES (CCL5) was used via Multiplex Electro-chemiluminescence (Meso Scale Discovery, USA) as per manufacturer's instructions.

### Rabbit Vaginal Irritation (RVI) Model

#### Treatment

Young adult reproductive age nulliparous Belgium white rabbits (5–8 months old, body weight 2.2 kg ± 20%) (*n* = 5 per group) were divided into rfhSP-D, placebo and SDS (positive control) groups. The aqueous gel formulation was prepared by dissolving methyl paraben 0.18% (w/v) and propyl paraben 0.02% (w/v) in heated glycerin 8.6% (v/v). Hydroxyethyl cellulose 2.5% (w/v) was added and dispersed to form an organic phase. Citric acid 1.0% (w/v) was dissolved in purified water alone or aqueous solutions of rfhSP-D (100 μg/ml) or 1% SDS. The pH was adjusted to 4.4, and the solution was clarified by passage through a 0.22 μm filter. Aqueous and organic phases were mixed and stirred well before use. Various gel formulations (1 ml) were administered intra-vaginally to their respective groups using an insulin syringe (without a needle) daily for 10 consecutive days. Necropsy was done on day 11 following euthanasia. The vaginal tissues were collected in formalin and processed for making paraffin blocks. 5μ ribbon of paraffin bearing sections were made using a microtome and collected on poly-L-lysine-coated glass slides.

#### RVI Scoring

RVI scoring of hematoxylin-eosin stained tissue slides was blinded. Briefly, the tissues were scored from 0 to 4 for epithelial damage (0 = normal, 1 = flattening, 2 = metaplasia, 3 = erosion, and 4 = ulceration) and leukocyte infiltration, edema and congestion (0 = absent, 1 = minimal, 3 = moderate, 4 = marked). At least three sections of vaginal tissues (both proximal and distal) of each animal were assessed for each of the above four parameters. Total score of each animal was calculated and was averaged with number of sections analyzed. Standard RVI method suggests that a total score from 1 to 4 is to be considered as minimal irritation, 5–8 as mild irritation, 9–11 as moderate irritation and 12–13 as marked irritation (41).

### Statistical Analysis

Student *t*-test, One-way analysis of variance (ANOVA; Bonferroni or Dunnett's multiple-comparison analyses) was performed using GraphPad Prism version 6.00 for Windows (GraphPad Software, San Diego, CA). *p*-value of <0.05 was considered significant.

## Results

### Global Gene Signature of HIV-1 Challenged EpiVaginal Tissues: Clues to Early Events During Vaginal Transmission

**Figure 2:**
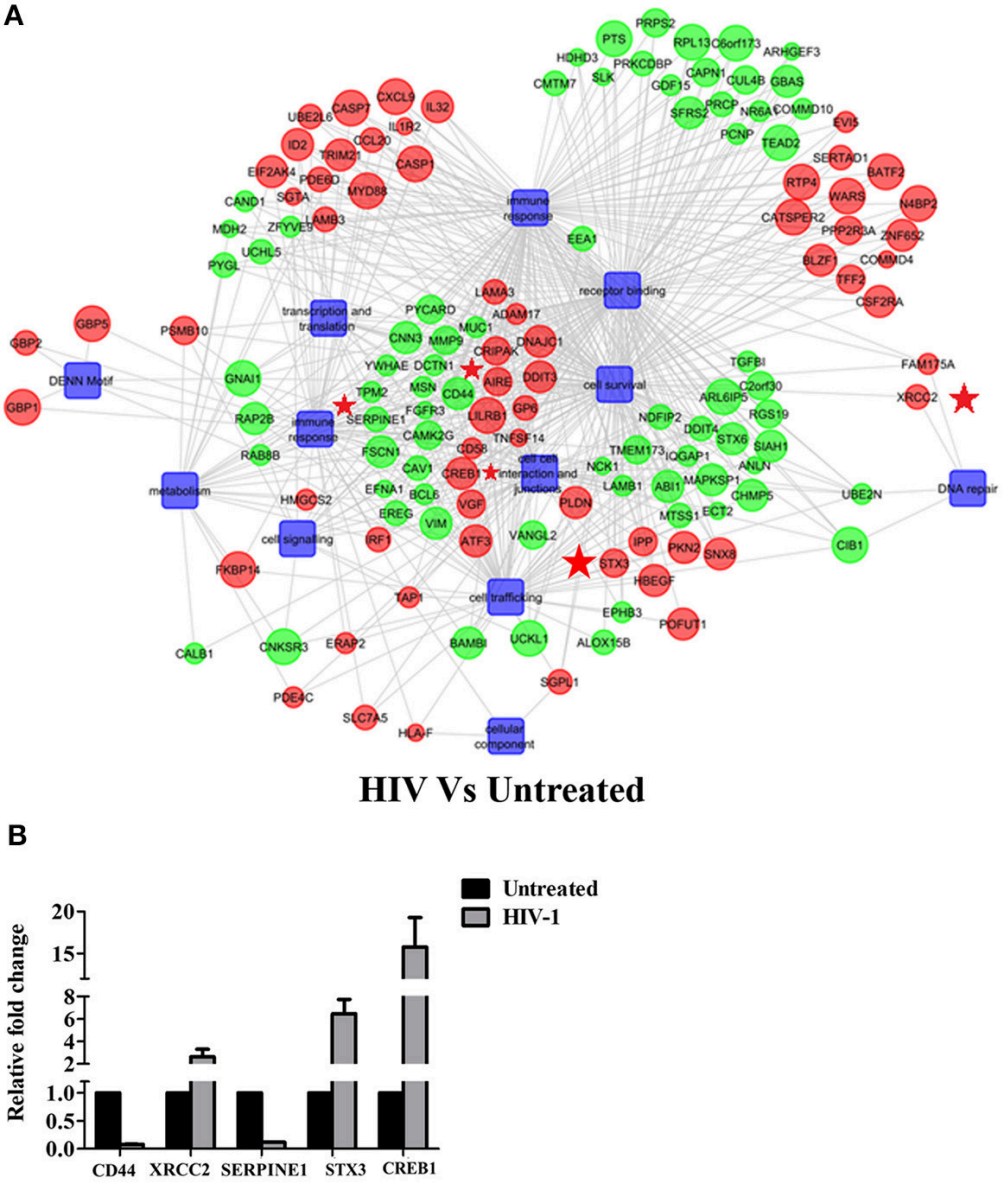
HIV-1 induced global gene signature of EpiVaginal tissues: **(A)** Gene regulatory network of differentially expressed genes and pathways by the EpiVaginal tissues treated with HIV-1 vs. Untreated EpiVaginal Control tissues. Biological processes are blue colored blocks downregulated genes are green colored, and upregulated are in red. Circles are sized according to their *p*-value. Genes that were used for validation (CD44, XRCC2, SERPINE1, STX3, CREB1) have been highlighted with a red star in their vicinity. **(B)** Validation of microarray data by real time qPCR. Ect/E6E7 cells were subjected to identical conditions and treatments as for EpiVaginal tissues. RNA was isolated and cDNA was subjected to real time qPCR. Data represents mean ± S. D of three independent experiments. Fold change in expression of the 5 genes for validation were statistically significant (*p* < 0.05) relative to untreated Ect/E6E7 cells.

Figure 1 depicts the experimental design used in the study to map the transcriptome of EpiVaginal tissues under different conditions. We report here, for the first time, a compendium of genes that were differentially expressed in EpiVaginal tissues when challenged with HIV-1 alone (355), HIV-1 in presence of rfhSP-D (518), or rfhSP-D alone (185) (Supplementary Figures S1, S2, S4). For the identification of differentially expressed genes, data was subjected to unsupervised hierarchical clustering using Pearson Uncentered algorithm with average linkage rule using Cluster 3.0 software. The resultant cluster was visualized using Tree View software. It revealed distinct patterns of upregulated and downregulated genes following treatments and indicated significant reproducibility within the replicates (Supplementary Figures S1, S2, S4). The microarray data was validated by evaluation of transcript expression by real time RT-PCR for six randomly selected, differentially expressed genes by HIV-1 and rfhSP-D to represent the three functional categories (Figures 2B, **4D**). Gene regulatory network analysis of the three interactions revealed involvement of several biological processes and pathways. Differentially expressed genes, along with the pathways, were subjected to regulatory network modeling, that resulting in the identification of key genes that act as bridges (involved in more than one process) and islands (which are specific to a process) (Figures 2A, **4B,C**, **6B**; Supplementary Figure S3). We have focused on three important processes involved in HIV-1 transmission, which are cell-cell interaction and barrier integrity, innate immune response, and cell survival. The networks were refined further to comprise the most relevant genes (**Figures 4B,C**, **6B**).

The gene signature of EpiVaginal tissues post 24 h HIV-1 exposure showed 187 upregulated and 168 downregulated genes (Supplementary Figure S1), associated with biological processes, such as cell integrity, inflammation and innate immune response, pyroptosis, cell survival, cell signaling and cytoskeleton (Figure 2A). Microarray results were validated by real time RT-PCR for PYCARD, CD44, XRCC2, SERPINE1, STX3, and CREB1 (Figure 2B). PYCARD, CD44, and SERPINE1 were among the prominent genes downregulated by HIV-1 but upregulated by rfhSP-D and XRCC2; STX3 and CREB1 were among the prominent genes upregulated by HIV-1 but downregulated by rfhSP-D.

HIV-1 induces a cytokine/chemokine storm at the mucosal sites (42) that facilitates the viral entry and transmission. HIV-1 challenged EpiVaginal tissues showed an upregulation of transcripts of cytokines and chemokines, such as IL-32, CCL20, and CXCL9. Transcripts of other pro-inflammatory genes, such as MYD88, ADAM17, TNFSF14, IL-1R2, HLA-F, CD58, PKN2, and STX3 were also upregulated. A significant upregulation of PSMB10, executioner caspases CASP7 and CASP1 of the inflammasome is suggestive of pyroptosis. However, a few inflammation-related genes MMP9, MUC-1, SERPINE1, TGF-α, TMEM173 were downregulated.

Interestingly, a group of interferon-inducible guanylate-binding proteins (GBP1, GBP2, and GBP5) were upregulated, suggesting that the vaginal epithelium attempts to mount an anti-viral response. TRIM21, another interferon-inducible gene, was also found to be upregulated along with the interferon-inducible transcription factors, such as IRF1, ATF3, BATF2, and CREB1.

A likely breach in the vaginal barrier after HIV-1 exposure is evident by alterations in several genes encoding for proteins of plasma membrane, cytoskeletal framework and gap junction. Actin cytoskeleton rearrangement (CNN3), integral plasma membrane proteins (CAV-1, STX6), microtubules and cytoplasmic dynactin binding (DCTN1), extracellular matrix glycoprotein (LAMB1), cell-cell recognition and signaling molecules (MSN, CD44) were all downregulated. Gap junction proteins, GJA1 and GJB6, were also downregulated. TFF2, which protects the mucosa from injury or insults, stabilizes the mucus layer and aids healing of the epithelium, was upregulated, suggesting a plausible feedback in response to the damaged epithelium.

**Figure 3:**
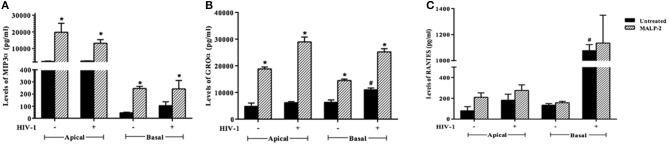
Chemokine response of EpiVaginal tissues upon HIV-1 challenge: Levels of **(A)** MIP-3α, **(B)** GRO-α, and **(C)** RANTES in the culture supernatant of EpiVaginal tissues (apical) and PBMCs (basal) with or without HIV-1 challenge. Each bar represents the mean ± S.D in pg/ml and of triplicates. ^*^indicates statistical significance (*p* < 0.05) relative to control (untreated) whereas # indicates statistical significance (*p* < 0.05) relative to HIV-1 challenge EpiVaginal tissues.

To corroborate the HIV-1 induced inflammation in the *ex vivo* model, we evaluated the levels of chemokines, such as RANTES, MIP-3α, and GRO-α in the apical (EpiVaginal) and basal (PBMCs) supernatants. A significant rise in RANTES and GRO-α but not in MIP-3α levels was observed in the supernatants of basal chambers of the EpiVaginal tissues upon HIV-1 challenge. MALP-2, a TLR2/6 agonist, induced all the three chemokines, indicating excessive inflammation (Figure 3), which is reminiscent of enhanced HIV-1 acquisition during RTI/STIs.

### HIV-1 Traverses Through EpiVaginal Tissue and rfhSP-D Impedes This Movement

The *ex vivo* model (Figure 1A). Mimics several aspects of vaginal transmission of HIV-1. The reconstructed, multi-layered EpiVaginal explants in the upper chamber serve as the first line of protection. The activated PBMCs present in the lower chamber serve as targets for the viral particles that traversed through the EpiVaginal tissues. In this model, along with HIV-1, rfhSP-D or both, we also used MALP-2 as a positive control for inflammation.

**Figure 4:**
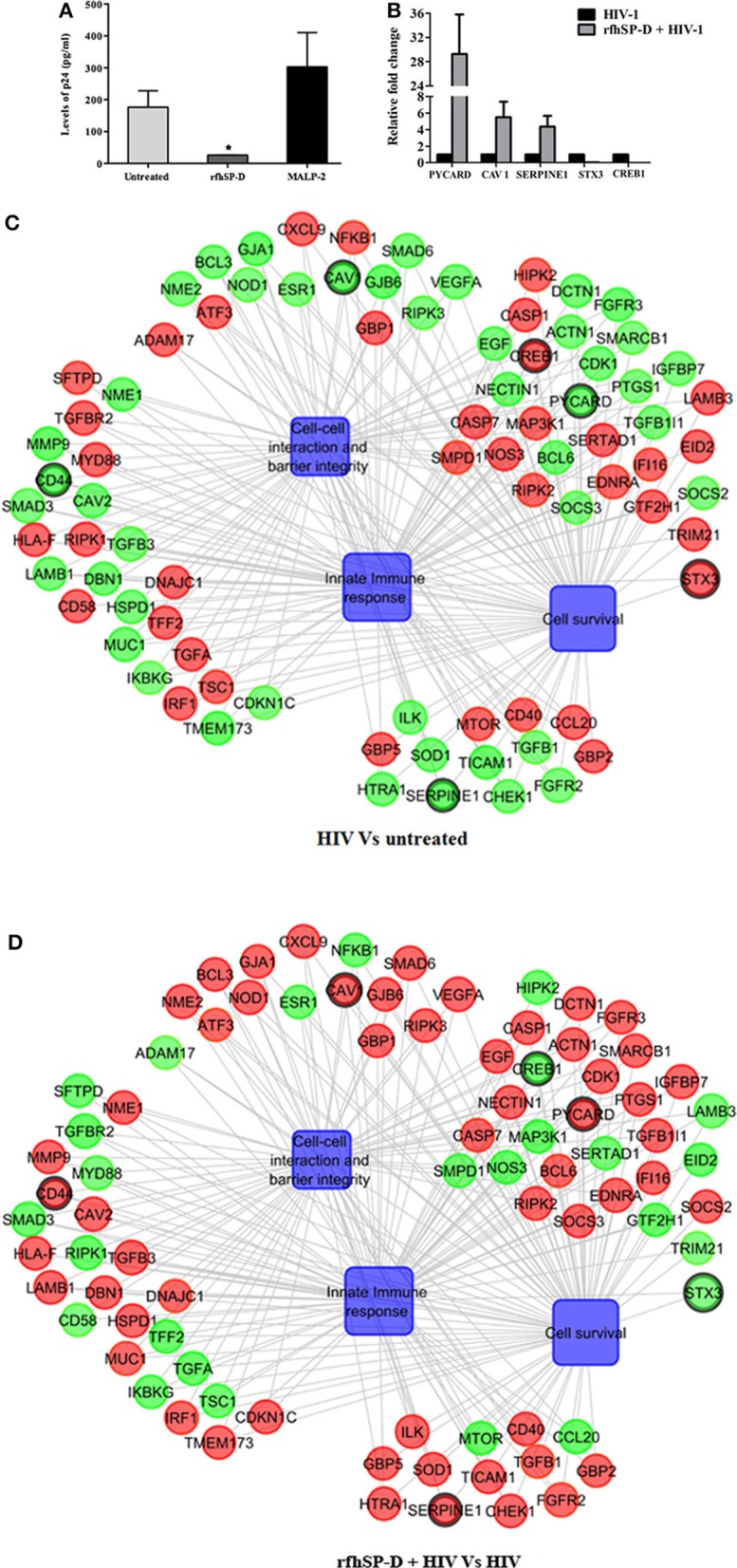
rfhSP-D impedes viral movement across the EpiVaginal tissue barrier and reverses HIV-1 induced gene signature: **(A)** Determination of HIV-1 p24 Ag by ELISA in supernatants from basal chambers at 24 h. Data represents mean ± S. D of three sets. ^*^indicates statistical significance ^*^p < 0.05 relative to medium alone. Gene regulatory network for EpiVaginal tissues treated with HIV-1 Vs untreated **(C)** and EpiVaginal tissues treated with rfhSP-D + HIV-1 vs. HIV-1 **(D)**. Biological processes are blue colored blocks and > 1.5-fold downregulated genes are green colored, > 1.5-fold upregulated genes are red, and <1.5-fold upregulated genes are orange. Circles are sized according to their *p*-value. **(B)** Validation of microarray data by real time RT-PCR. Ect/E6E7 cells were subjected to identical conditions and treatments as for EpiVaginal tissues. RNA was isolated and cDNA was subjected to real time RT-PCR. Data represents mean ± S. D of three independent experiments. Fold change in expression of the 5 genes for validation were statistically significant (*p* < 0.05) relative to untreated Ect/E6E7 cells.

At 24 h, HIV-1 p24 Ag was detected in the supernatants of basal chambers. A higher level of p24 Ag detected in the supernatants, when the vaginal tissues were challenged with HIV-1 in presence of MALP-2 (>1.6-fold higher than control; [Medium alone]), suggested that more virions migrated to the basal chamber (though could not attain statistical significance). RfhSP-D significantly reduced the viral transfer and only one-fifth (20 ± 2.6%) of the HIV-1 p24 Ag level was detected in the basal PBMCs supernatants, as compared to the HIV-1 alone (100%) (Figure 4A).

### RfhSP-D Reverses the HIV-1-Induced Gene Signature: Decoding the Protective Response

To recognize the gene signature that illustrates inhibition of vaginal transfer of HIV-1, we analyzed transcriptome of rfhSP-D treated HIV-1 challenged EpiVaginal tissues in the apical chamber (Supplementary Figure S2). We observed a remarkable reversal of gene expression associated with gap junction proteins, plasma membrane and cytoskeletal framework of the cell. Several genes including GJA1, GJB6, CAV1, CAV2, LAMB1, ACTN1, DBN1, and DCTN1 were upregulated upon rfhSP-D treatment, which were otherwise downregulated by HIV-1 (Supplementary Table S2; Figures 4B,C). Maintenance of vaginal barrier integrity by rfhSP-D was evident from upregulation of NECTIN1 and CD44 along with gap junction genes. Interestingly, DBN1, ACTN1, NECTIN1, and CD44 have been shown to act as anti-viral or entry inhibitors (Table 1). Inflammation is the primary reason for epithelium breakage and compromised vaginal barrier. RfhSP-D reversed HIV-1 induced inflammatory genes, such as ADAM17, MYD88, SMAD3, SMAD6, CD58, CCL20, TRIM21, and SMARC1. NF-κB and mTOR, the two master regulators of inflammation, were also downregulated, suggesting induction of a state of quiescence within the EpiVaginal tissues. A few anti-inflammatory genes were upregulated; IL-20, BCL-3, NME1, NME2, CHEK1, and CDKN1C. SOCS2 and SOCS3 were selectively upregulated, suggesting rfhSP-D mediated dampening of pro-inflammatory cytokine production. TGF-β pathway seems to be relevant in vaginal transfer of virus since several genes (SMAD3, SMAD6, TSC1, EID2, TGF-β-1L1, TGF-βR, and TGFA) of this pathway were altered by HIV-1 and reversed by rfhSP-D (Table 1; Figures 4C,D).

**Table 1:** rfhSP-D-mediated reversal of HIV-1 induced alteration of gene expressions in EpiVaginal tissues.

**Gene name**	**HIV-1 (FC)**	**rfhSP-D + HIV-1 (FC)**	**Functions**	**Ref**.	**Role in HIV**	**Ref**.
**INFLAMMATION**						
ADAM17	Up (1.51)	Down (−1.16)	A protease critical in cleavage of TNF-α and other inflammatory proteins to active form. Important in diverse cellular processes such proliferation, migration, cell adhesion	PMID: 20184396	Nef activates and shuttles activated ADAM17 into exosomes Exosomal Nef and ADAM17 activates quiescent CD4+ T Lymphocytes via TNF-α	PMID: 23317503PMC4178784
MMP9	Down (−3.69)	Up (3.75)	Proteolytic enzyme, degrades extracellular matrix.	PMID: 12540195	Induced by Tat in astrocytes Upregulated by gp120 in vaginal epithelial cell line	PMC2679334 PMC3222676
MYD88	Up (1.82)	Down (−1.26)	universal adapter protein downstram of TLRs (except TLR 3) to activate the transcription factor NF-κB	PMID 18064347	HIV-1 Tat Activates both the MyD88 and TRIF Pathways To Induce TNF-α and IL-10 in Monocytes	PMID: 27053552
RIPK1	Up (1.29)	Down (−1.95)	Serine/threonine kinase that regulate a variety of cellular processes such as cell death and innate immune responses to viral and bacterial infection, induces necroptosis	PMID: 19524512 PMID: 24129419 PMID: 26086143	Cleaved by HIV proteases and modulate cellular response	PMC4546280
CD58	Up (1.64)	Down (−1.25)	Interaction between CD2 and its counterreceptor, CD58 (LFA-3) aids in T cell-APC cell cell contact	PMID: 10380930	Engagement of CD58 enhances HIV-1 replication in monocytic cells	PMID: 8656013
TFF2	Up (1.99)	Down (−2.11)	Secreted into the mucus layer where it stabilizes the mucin gel layer and stimulates migration of epithelial cells. Upgregulated in chronic inflammation	PMID: 19064997	–	–
SERPINE1	Down (−2.04)	Up (2.92)	An inhibitor of fibrinolysis, high concentrations of the gene product are associated with thrombophilia	PMID: 24669362	Monocytes from asymptomatic viremic HIV(+) individuals show increased PAI-1 (SERPINE1)	PMID: 22815948
CCL20	Up (2.94)	Down (−2.61)	Responsible for the chemo-attraction of iDCs, effector/memory B cells and T cells. High specificity for CCR6	PMID: 27617163	Attracting key immune cells, including Th17 cells and dendritic cells, to sites of infection and propagating the virus to other sites of the body	PMID: 28005525
TRIM21	Up (3.29)	No change (1.08)	Intracellular antibody effector in the intracellular antibody-mediated proteolysis pathway. Directs the virions to the proteasome.	PMID: 21045130	Chimeric restriction factor TRIM21-CypA provides highly potent protection against HIV-1 without loss of normal innate immune TRIM activity	PMID: 22909012
SOCS2	Down (−1.2)	Up (1.63)	Down-regulation of cytokine signaling	PMID: 12208853	Tat impaired the IFN γ - receptor signaling pathway at the level of STAT1 activation, via Tat-dependent induction of suppressor of cytokine signaling-2 (SOCS-2) activity	PMID: 19279332
SOCS3	No change (1.09)	Up (1.9)	Down-regulation of cytokine signaling	PMID: 9202125 PMID: 9430658 PMID: 9857039	Protein levels were lower in CD4 (+) T cells of HIV-infected patients than in healthy controls, Suppressed Th17 levels correlate with elevated SOCS3 expression in CD4 T cells during acute simian immunodeficiency virus infection	PMID: 21337543 PMID: 23596301
NOS3	Up (1.16)	Down (−1.14)	Major determinant of vascular tone and blood pressure	PMID: 7514568	Nitric oxide inhibits HIV tat-induced NF-κB activation	PMID: 10393859
PYCARD	Down (−1.64)	Up (2.47)	Involved in NLRP3 induced inflammasome. Responsible for cleavage of pro-caspase 1	PMID: 20303873	Involved caspase-1 dependent pyroptosis of HIV infected CD4 T cells	PMC4047036
SMARCD1	Down (−1.95)	Up (2.45)	Part of SWI/SNF complexes that regulate gene activity of chromatin remodeling, may act as tumor suppressor	PMCID: PMC5406539	Role in HIV-1 assembly, interaction between Nef and INI1/SMARCB1 augments replicability of HIV-1 in resting PBMCs facilitate Tat-mediated HIV-1 transcription	PMID: 27558426 PMID: 25559666 PMID: 16889668
CREB1	Up (1.69)	Down (−2.04)	CREB family of transcription factors consists of cAMP-responsive activators including CREB, cAMP response element modulator, and activating transcription factor	PMID: 10872467	Tat utilizes CREB to promote IL-10 production, although the significance of this regarding HIV pathogenesis is not entirely clear, IL-10 can inhibit HIV-1 replication in monocytes and macrophages	PMID: 7527449
RIPK3	Down (−1.28)	Up (1.64)	Serine/threonine kinases that regulate a cellular processes such as cell death and innate immune responses to viral and bacterial infection, induces necroptosis	PMID: 19524512 PMID: 24129419 PMID: 26086143	Not cleaved by HIV proteases and modulate cellular response	PMC4546280
SOD1	Down (−1.30)	Up (1.23)	Enzyme attaches (binds) to molecules of copper and zinc to break down toxic, charged oxygen molecules called superoxide radicals.	PMID: 7901908	SOD1 prevented gp120 and Tat elicited reactive oxygen species (ROS) and rescued neuron apoptosis	PMID: 17336361
TGFBR2	Up (1.21)	Down (−1.7)	TGF-β mediates its actions through heteromeric kinase receptor complex consisting of TGF receptors of type 1 and 2	PMID: 1333888	Increased expression upon Tat treatment of epithelial cells	PMID: 15857508
TGFA	Up (1.15)	Down (−1.46)	Exerts several effects on target cells, such as neovascularization promotion and mitogenic signaling.	PMID: 9242560	Significant rise in chronic HIV type 1 infection	PMID: 27268396
SMAD6	Down (−1.37)	Up(1.59)	Smad6 inhibits signaling by the TGF-beta superfamily	PMID: 9335505	Down-regulated after Tat treatment of U937 macrophages	PMID: 16282533
STX3	Up (2.01)	Down (−2.04)	Potentially involved in secretion of IL-6 from dendritic cells following activation of TLRs	PMID: 25674084	Depletion of STX3 reduced HCMV production	PMID: 25583387
XRCC2	Up (2.33)	Down (−2.38)	DNA repair protein binding to double stranded breaks	PMID: 10227297	Suppression of retroviral infection by XRCC2	PMID: 15297876
**CYTOSKELETON AND CELL-CELL INTERACTION AND INTEGRITY**						
GJA1	Down (−3.07)	Up (3.39)	Involved in intercellular communication (GJIC) between cells to regulate cell death, proliferation, and differentiation. Involved in inflammation	PMID: 25110696 PMID: 25560303	–	–
CD44	Down (−2.42)	Up (5.25)	Cell-surface glycoprotein involved in cell–cell interactions, cell adhesion and migration	PMID: 28546458	Blocking of HIV entry through CD44–hyaluronic acid interactions.	PMID: 25155464 PMID: 25320329
CAV1	Down (−1.89)	Up (2.53)	Cav-1 is enriched in caveolae, involved in endocytosis, signal transduction. Role in innate immune defense, and it regulates macrophage cytokine production and signaling	PMID: 16982844	Cav-1Tat induced alterations of tight junction protein. Cav-1 mediated uptake via langerin restricts HIV-1 infectivity	PMID: 18667611PMID: 25551286
CAV2	Down (−1.22)	Up (2.48)	Similar to Cav-1 and also inhibits cell proliferation, migration and invasion	PMID: 23454155	–	–
DBN1	Down (−1.86)	Up (1.74)	DBN1 suppresses dynamin-mediated endocytosis via interaction with cortactin. DBN1 restricts the entry of viruses into host cells and more broadly to function as a crucial negative regulator of diverse dynamin-dependent endocytic pathways	PMID: 28416666	Drebrin is a negative regulator of HIV entry and HIV-mediated cell fusion. Down-regulation of drebrin expression promotes HIV-1 entry, decreases F-actin polymerization, and enhances profilin local accumulation in response to HIV-1	PMID: 23926103
NECTIN1	No change (1.04)	Up (2.94)	Nectin cell adhesion molecule, plays role in organization of adheren junctions and tight junction	PMID: 28392352	HIV-Induced Exposure of Nectin-1 Facilitates HSV-1 Infection	PMID: 24586397
IGFBP3	No change (−1.05)	Up (2.11)	Binds IGF-I and IGF-II with relatively low affinity, and belongs to a subfamily of low-affinity IGFBPs. It also stimulates prostacyclin production and cell adhesion.	PMID: 21835307	Inhibit the replication of HIV-1 in cultured cord blood mononuclear cells and chronically HIV-infected U937 cells	PMID: 7576911
ACTN1	No change (−1.01)	Up (1.34)	Major actin cross-linking proteins found in virtually all cell types as a cytoskeleton.	PMID: 26312134	α-Actinin regulates the immune synapse formation and is required for efficient T cell activation. silencing of either EWI-2 or α-actinin-4 increased cell infectivity. Regulation of the actin cytoskeleton at T cell immune and virological synapses	PMID: 22689882
GJB6	Down (−1.95)	Up (2.87)	Gap junctions allow the transport of ions and metabolites between the cytoplasm of adjacent cells	PMID: 19944606	Gap junction channels shutdown under inflammatory conditions, including viral diseases.	PMC4774036

*FC-Fold Change*.

We identified some pro-inflammatory genes that were upregulated in rfhSP-D treated HIV-challenged EpiVaginal tissues, such as CD40, ILK, ESR1, EGF, FGFR2, HIPK2, SOD1, MAP3K1, IFI16, NOD2, IL-1B, HTRA1, EDNRA. Interestingly, there was a downregulation of the intrinsic SP-D gene expression that suggested a mitigation of inflammatory response of vaginal tissues in presence of rfhSP-D (Figures 4C,D). HIV-1 challenged EpiVaginal tissues showed an upregulation of SFTPD (gene encoding SP-D protein) transcript (Figure 4C) whereas, rfhSP-D pre-treatment followed by HIV-1 challenge reverted the HIV-1 induced upregulation of rfhSP-D (Figure 4D). rfhSP-D treatment alone did not alter the expression of native SP-D transcript (**Figure 6B**).

### HIV-1 Induced Downregulation of Tight Junction Gene Expression Is Rescued by RfhSP-D

**Figure 5:**
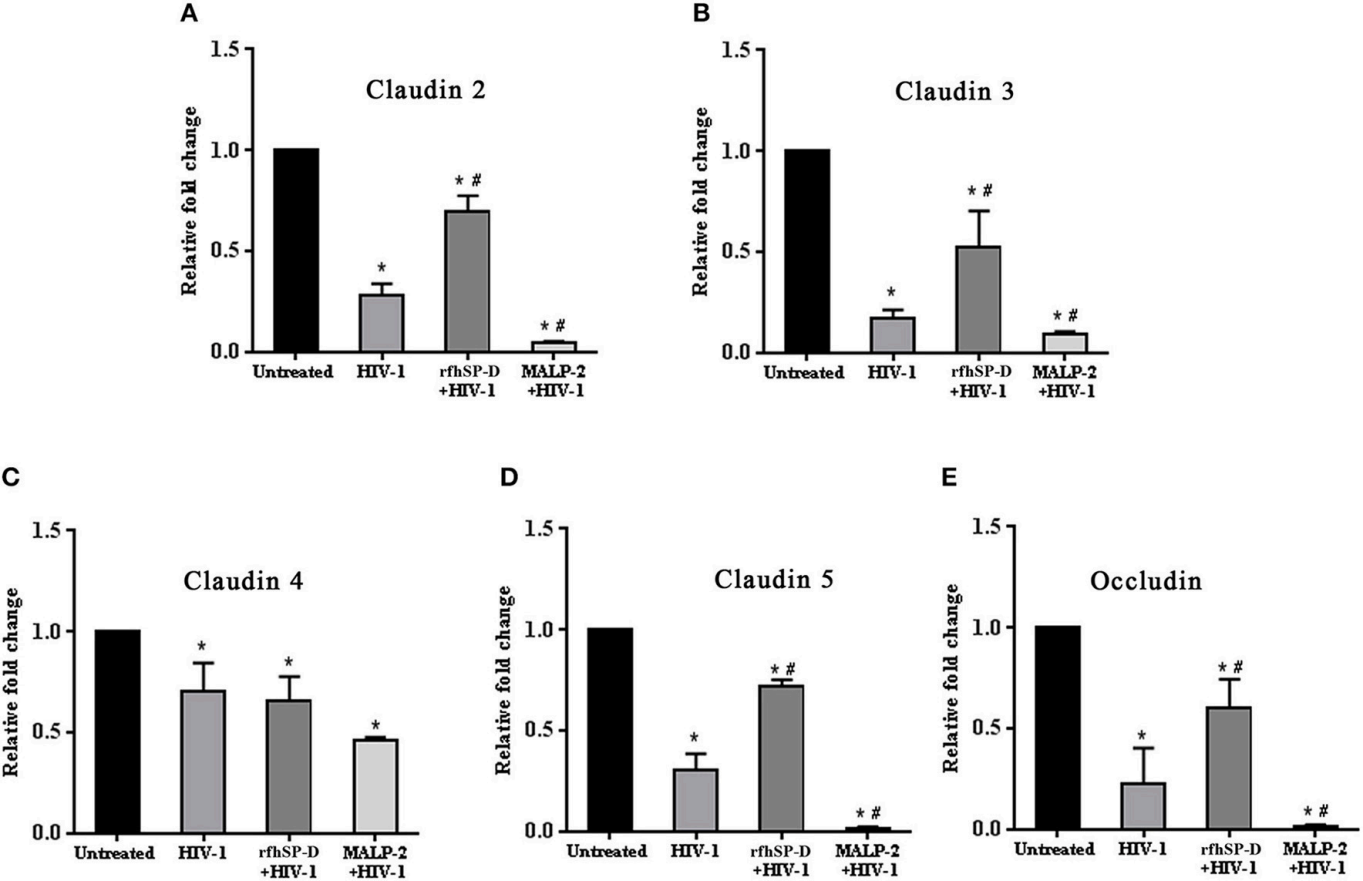
HIV-1 mediated downregulation of transcripts of tight junction genes while rfhSP-D maintained integrity. Relative expression of transcripts of **(A)** Claudin 2, **(B)** Claudin 3, **(C)** Claudin 4, **(D)** Claudin 5, **(E)** Occludin were determined in EpiVaginal tissues by real time qPCR. When compared with HIV-1 challenged tissues, rfhSP-D treatment led to significant upregulation of Claudin 2, 3, 5, and occludin. Data is represented as mean ± S. D. ^*^, # indicate statistical significance of *p* < 0.05 in comparison to untreated and HIV-1 treated EpiVaginal tissues, respectively.

HIV-1 is known to downregulate tight junction proteins in order to traverse through the weakened vaginal barrier (36). We assessed the status of claudins and occludin in the EpiVaginal tissues. HIV-1 challenge led to a significant decrease in the transcript levels of tight junction proteins that were further downregulated when simultaneously treated with MALP-2 (Figures 5A–E). RfhSP-D countered the HIV-1 induced downregulation of transcripts of claudin 2, 3, 5 and occludin, except claudin 4, suggesting a reduced damage to the vaginal integrity (Figures 5A–E).

### RfhSP-D Does Not Enhance the Susceptibility of Target Cells to HIV-1 Acquisition

**Figure 6:**
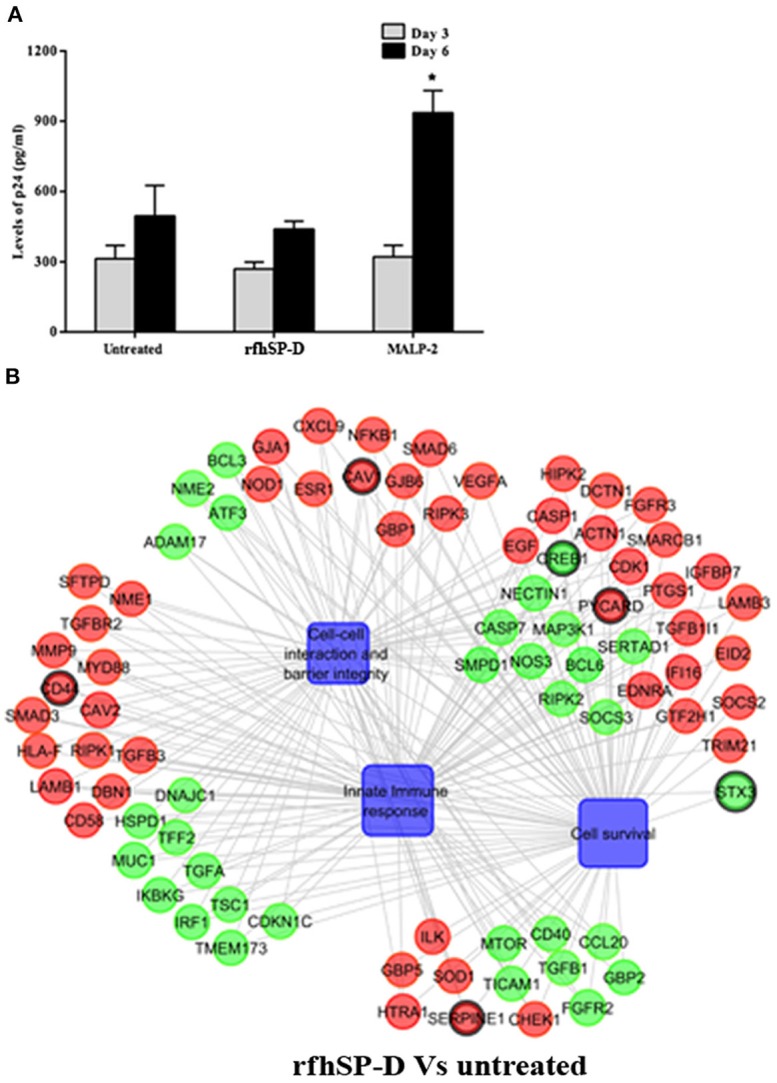
Susceptibility of PBMCs to HIV-1 acquisition: **(A)** Day 3 and 6 HIV-1 p24 Ag levels as determined by ELISA. RfhSP-D did not enhance susceptibility to HIV acquisition whereas MALP-2 treatment enhanced it on day 6. Data is representative of three biological replicates and is represented as mean ± S. D. ^*^indicate statistical significance of *p* < 0.05 in comparison to day 6 untreated (control). **(B)** Gene regulatory network for EpiVaginal tissues treated with rfhSP-D vs. untreated. Biological processes are blue colored blocks, downregulated genes are green colored, upregulated are red, unaltered are in orange. Circles are sized according to their *p*-value.

Another experimental setup was designed to interrogate whether rfhSP-D, on its own, caused inflammation within EpiVaginal tissues (in the absence of HIV-1 challenge), which in turn increased susceptibility of target cells (Figure 1B). PBMCs in the basal chamber of rfhSP-D treated tissues showed no significant increase in the acquisition of HIV-1 while the inflammatory MALP-2 treated tissues showed increased p24 levels on day 6 (Figure 6A).

### RfhSP-D Treatment Strengthened Vaginal Barrier: SP-D a Natural Vaginal Host Defense Molecule

Alterations in the transcripts of EpiVaginal tissues induced by rfhSP-D were also identified by microarray analysis. Of the total 185 genes differentially regulated, 103 were upregulated and 82 were downregulated (Supplementary Figure S4). Upregulation of CAV1 and CAV2, along with Laminins (LAMA3, LAMB1, LAMC1, and LAMC2), which are essential for formation and function of the basement membrane, was suggestive of a strengthened mucosal barrier. Collagen transcripts (COL4A5, COL5A1, COL5A2 and COL7A1, COL17A1), important structural components of basement membranes, were also upregulated. With an integral role in adhesion of the epithelium to extracellular matrix, integrins α_3_, α_6_, and β_4_, were upregulated by rfhSP-D (Figure 6B; Supplementary Figure S5). Specific upregulation of genes related to structural stability of the cell and epithelial integrity suggested that rfhSP-D strengthened the local tissue architecture. Consistent with its established anti-inflammatory role, rfhSP-D downregulated genes that promote inflammatory signals, such as GBP2, IRF1, ATF3, CREB1, IGFBP2, and IGFBP7.

SP-D is synthesized by the human vaginal epithelial cells and its uterine expression is hormone regulated (17). We detected SP-D in the vaginal lavage of normal cycling women (Supplementary Figure S5A). Vaginal epithelial cells (Vk2/E6E7) also showed transcripts of SP-D (Supplementary Figure S5B). In addition, confocal microscopy revealed that SP-D protein was being produced by Vk2/E6E7 and could be localized in the cytoplasm (Supplementary Figure S5C). With its natural presence in the vaginal tract, its role as a pattern recognition protein and in strengthening of the vaginal barrier as evident from gene expression studies, SP-D seems to be vital as the first line of defense at the vaginal surfaces.

### rfhSP-D Has No Adverse Effect on Cell Viability and NF-κB Translocation

**Figure 7:**
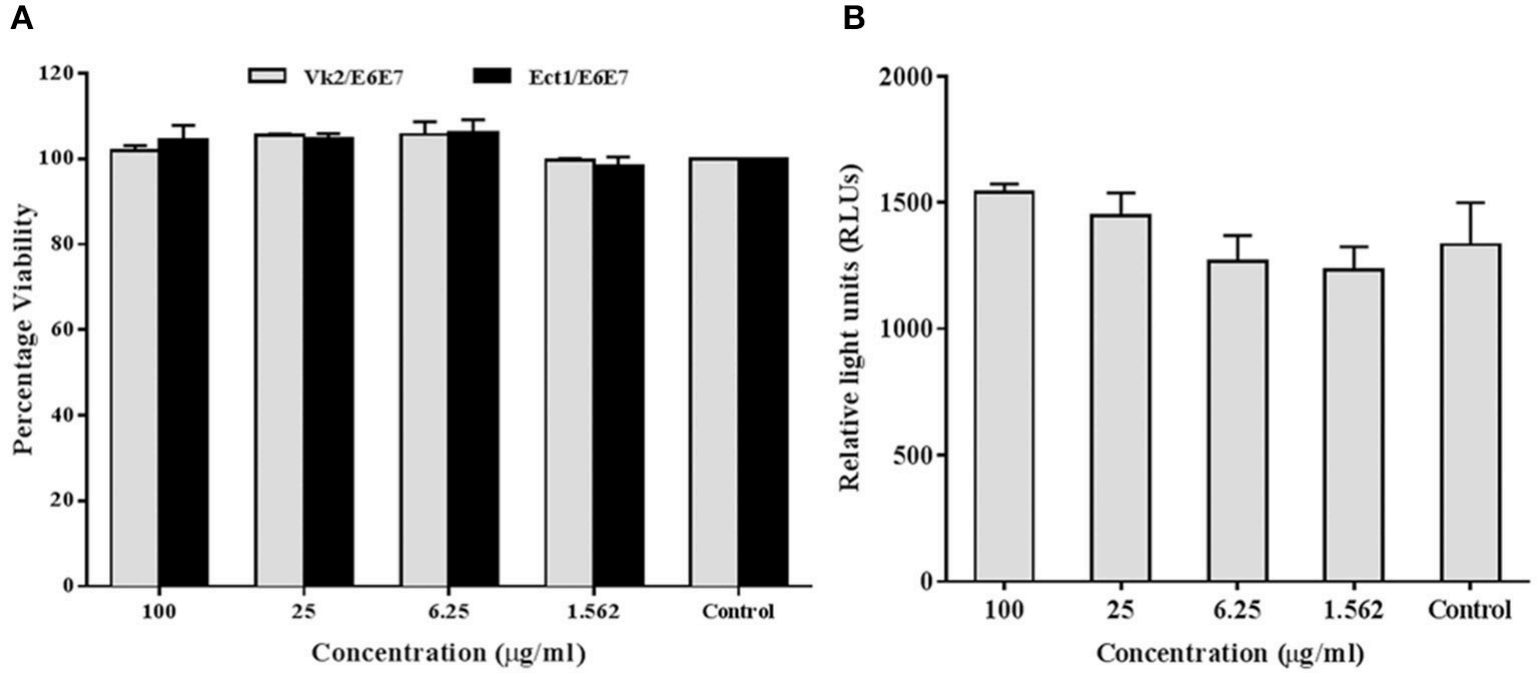
rfhSP-D does not affect viability or NF-κB activation: **(A)** MTT assay showing no significant alteration in cellular viability of vaginal (Vk2/E6E7) and ectocervical (Ect1/E6E7) cells 24 h after rfhSP-D treatment. **(B)** NF-κB activity measured by firefly luciferase reporter assay at 24 h of stimulation of endocervical epithelial (End1/NF-κB) cells with rfhSP-D (up to 100 μg/ml). Values represent mean ± SD.

In addition to the *ex vivo* efficacy of rfhSP-D as an inhibitor of the vaginal transmission of HIV-1, it was pertinent to evaluate the safety of rfhSP-D application on the vaginal surface. As a first step, we assessed its effect on the viability and inflammation of vaginal and ectocervical cells. Within the concentration range of 1.562–100 μg/ml and a duration of 24 h treatment, the viability of vaginal and ectocervical cells was unaltered (Figure 7A). NF-κB activation is a prerequisite for inflammation and breach of vaginal barrier providing access to HIV-1 entry. Hence, to determine the effect of rfhSP-D on the NF-κB activation, we used an endocervical cell line (End1/E6E7) transfected with pHTS–NF-κB firefly luciferase reporter. None of the indicated rfhSP-D concentrations induced NF-κB activation, whereas MALP-2 and poly I:C, agonists of TLR-2/6 and TLR3 respectively, led to a significant activation (Figure 7B). Furthermore, rfhSP-D did not cause any alteration in the levels of anti-inflammatory immune mediators, such as interleukin-1 receptor antagonist (IL-1RA), secretory leukocyte protease inhibitor (SLPI) and elafin, which are known to maintain vaginal homeostasis (data not shown).

### RfhSP-D Does Not Adversely Affect Vaginal *Lactobacilli*

**Figure 8:**
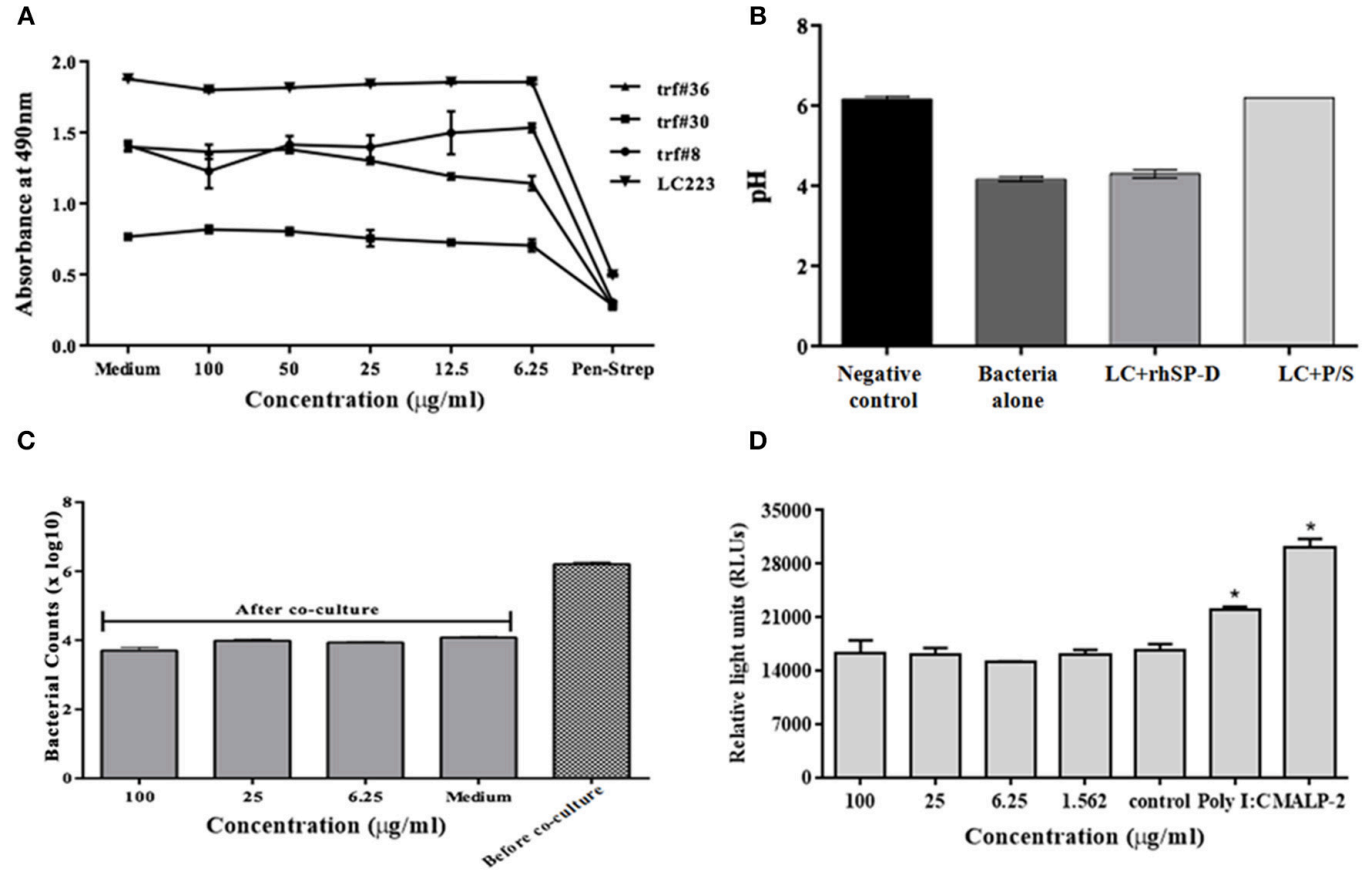
No adverse effects of rfhSP-D on vaginal lactobacilli and epithelium-commensal interaction: **(A)** Bacterial growth was assessed at 490 nm. At 24 h, none of the indicated rfhSP-D concentrations led to any alteration in growth of lactobacilli (*Lactobacillus fermentum spps mucosae* (TRF#36), *Lactobacillus gasseri* (TRF#8), *Lactobacillus salivarius* (TRF#30) and *Lactobacillus crispatus* (LC223) whereas penicillin-streptomycin (P/S) significantly inhibited its growth. **(B)** pH of the spent medium is a measure of lactic acid production. LC-Lactobacillus crispatus (LC223). Values represent means ± SD of three experiments. **(C)** CFU counts before and after epithelial (Vk2/E6E7)-bacterial (*Lactobacillus crispatus*) co-cultures were treated with rfhSP-D. **(D)** Co-cultures of epithelial cells (End1/NF-κB)-bacteria (*Lactobacillus crispatus*) were assayed for luciferase activity. No apparent rise in luciferase activity was observed following treatment with rfhSP-D whereas Poly I:C and MALP-2 showed a significant increase in NF-κB activity. Values represent mean ± SD. ^*^p < 0.05 in comparison to untreated.

*Lactobacilli*, as vaginal commensals, are integral to the female reproductive tract. A direct toxicity assay revealed that rfhSP-D did not adversely affect viability of the clinical isolates of *Lactobacilli* (TRF #8, TRF #30, TRF#36 and *Lactobacillus crispatus* LC223) (Figure 8A). Lactic acid produced by *Lactobacilli* contributes to vaginal defense and any alteration in its production would enhance susceptibility to pathogens including HIV-1 (43). The pH of the supernatant from cultures treated with rfhSP-D was acidic like untreated controls. As expected, Pen-Strep reduced the viability of *Lactobacilli* and the supernatant showed a significantly higher pH (toward neutral) (Figure 8B).

### rfhSP-D Does Not Interfere With Vaginal Epithelium-*Lactobacilli* Interaction

Since the vaginal microflora tightly controls the epithelial immune functions in a species- and strain-specific manner, any interference from topically applied microbicides or potential anti-HIV-1 agents may prove detrimental. Thus, we employed vaginal *Lactobacilli* colonization model that mimics *in vivo* conditions (6). In the co-culture conditions, rfhSP-D treatment did not lead to any reduction in CFU counts (Figure 8C).

**Figure 9:**
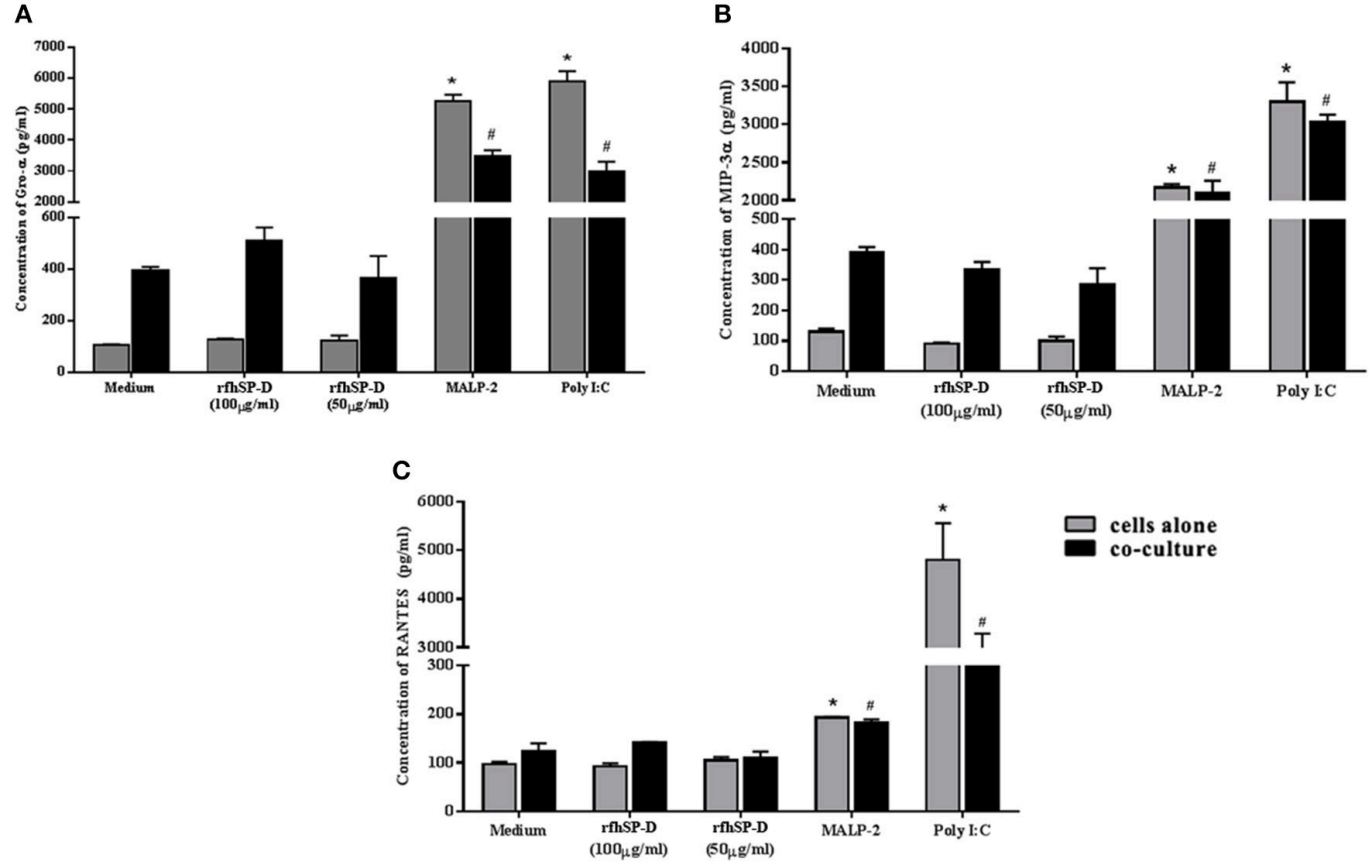
rfhSP-D does not alter basal levels of chemokines: **(A)** GRO-α, **(B)** MIP-3α, and **(C)** RANTES Levels were determined using MSD assay in the epithelial cells (Vk2/E6E7)—bacterial (*Lactobacillus crispatus*, LC223) co-culture. Data is representative as mean ± S. D. ^*^p < 0.05 was considered statistically significant.

Epithelial interaction with commensals leads to enhanced inflammation in a regulated manner; when exacerbated, it enhances susceptibility to HIV-1 and when calmed, it compromises immunity. Hence, we assessed the effect of rfhSP-D on NF-κB induction in this co-culture model. Importantly, NF-κB levels were not affected across all the tested concentrations of rfhSP-D (Figure 8D). Poly I:C and MALP-2 did show an exaggerated NF-κB activity. Further, rfhSP-D did not significantly alter the levels of chemokines, such as RANTES, GRO-α, MIP-3α, corroborating no adverse effect on vaginal immune physiology (Figures 9A–C).

### Repeated Application of rfhSP-D on Rabbit Vaginal Surface Does Not Induce Inflammation

**Figure 10:**
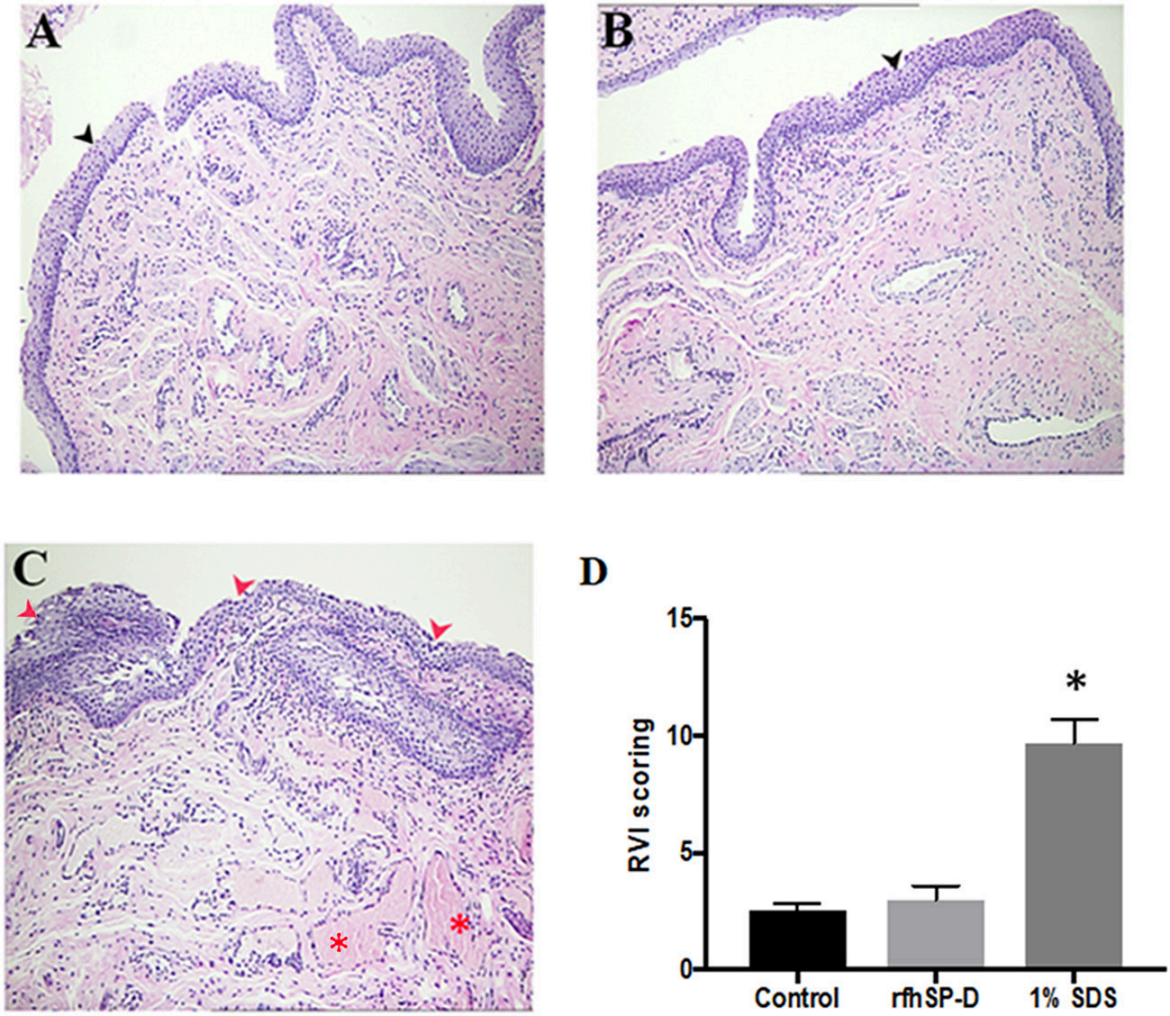
Rabbit Vaginal Irritation (RVI) model demonstrates intact integrity of mucosal barrier on repeated application of rfhSP-D gel: H&E staining of vaginal sections of rabbits (*n* = 5/group) treated with **(A)** placebo gel **(B)** rfhSP-D (100 μg/ml) gel and **(C)** 1% SDS gel (positive control) daily for 10 consecutive days. Sections from 1% SDS gel treated rabbits showed inflamed epithelial barrier with significant infiltration of polymorphonuclear cells (PMNs) (depicted by “red arrows”) and hemorrhage (depicted by the “red asterisk”). Black arrow heads depict epithelial membrane with minimal infiltration of PMNs in the “placebo gel” and “rfhSP-D (100 μg/mL) gel” in **(A,B)**. Magnification 10×. **(D)** RVI score of the rfhSP-D treated group was not significantly different from the placebo group. At least three sections of vaginal tissues (both proximal and distal) of each animal (blinded) were scored from 0 to 4 for epithelial damage (0 = normal, 1 = flattening, 2 = metaplasia, 3 = erosion, and 4 = ulceration) and leukocyte infiltration, edema and congestion (0 = absent, 1 = minimal, 3 = moderate, 4 = marked). Total score of each animal was calculated and was averaged with number of sections analyzed. A total score from 1 to 4 is to be considered as minimal irritation, 5–8 as mild irritation, 9–11 as moderate irritation, and 12–13 as marked irritation. ^*^p < 0.05 was considered statistically significant.

Rabbits with repeated vaginal application of 1% SDS (positive controls) showed rupturing of the epithelial barrier and hemorrhage, whereas, rfhSP-D and placebo groups showed no signs of inflammation (Figures 10A–C). As per the RVI scoring, vaginal sections of rfhSP-D and placebo groups showed none or minimal irritation. The total sum of RVI scoring was 2.98 ± 0.6 for the rfhSP-D group and was not significantly different from the RVI score of 2.54 ± 0.3 for the placebo group, whereas, 1% SDS showed a moderate inflammation score of 9.7 ± 1.01, indicating gross toxicity (Figure 10D).

## Discussion

Inflammation and breach of mucosal barrier are the two major events that render the “gatekeeping mechanisms” ineffective leading to HIV-1 transmission. The present study establishes a recombinant fragment of human SP-D (rfhSP-D) as a candidate microbicide, which remarkably inhibited HIV-1 transfer in an *ex vivo* model comprising of multi-layered vaginal mucosal tissue. We also report the gene expression profile of HIV-1 challenged EpiVaginal tissues. RfhSP-D specifically reversed the infection-promoting gene signature induced by the virus, thereby, maintaining the integrity of vaginal epithelium and suppressing the pro-inflammatory milieu. Furthermore, *in vitro* and *in vivo* safety studies implied that the rfhSP-D is safe for mucosal application at the concentrations that to restrict viral passage.

**Figure 11:**
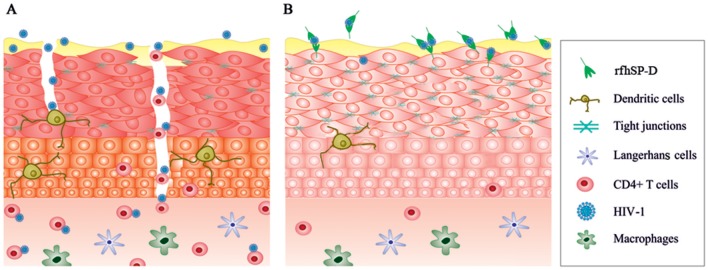
A schematic model illustrating effects of HIV-1 and rfhSP-D on EpiVaginal tissues. **(A)** The intact epithelium seems to be breached after HIV-1 exposure. Alterations in the genes encoding tight junction proteins, cytoskeleton and those contributing to inflammation are plausibly the critical events in HIV-1 transmission through the multi-layered tissue. **(B)** rfhSP-D potently binds to HIV-1 and interacts with EpiVaginal tissues, reverses HIV-1 induced gene signature, and inhibits HIV-1 transmission.

Transcriptome snapshot of the EpiVaginal tissues upon HIV-1 challenge revealed an inflammatory response comprising of chemokines, cytokines and components of inflammasome. Upregulation of these genes would act in sync, contributing to a generalized local inflammation in the vaginal epithelium. Fanibunda et al. (44) reported global gene expression in a monolayer of vaginal epithelial cell line (Vk2/E6E7) challenged with HIV-1 recombinant gp120 protein with a predominant induction of immunomodulatory processes and proteases. Following HIV-1 exposure, primary genital epithelial cell cultures showed enhanced proinflammatory cytokines (e.g., TNF-α and IL-6) and disruption of tight junctions, such as claudins, occluding, and ZO-1, leading to a compromised barrier (36, 45). Barouch et al. demonstrated that 24 h post-vaginal SIV challenge, the host lacked expression of the antiviral restriction factors and the response comprised of NLRX1 and TGF-α which incapacitated a strong anti-viral response (46). Consistent with the previous reports, the *ex vivo* model of human vaginal tissues showed pro-inflammatory response on viral challenge. Alongwith, it showed upregulation of host restriction factors, such as guanylate-binding proteins (GBP1, GBP2, GBP5), TRIM21 and other IFN-inducible genes. GBP5 has been recently reported as a host restriction factor in virus-challenged macrophages (47). Although, not proven in the context of HIV-1, TRIM21 is known to obstruct the incoming antibody-opsonized non-enveloped virions and efficiently mediate post-entry neutralization and innate immune signaling (48, 49). Being effective intracellularly, it is possible to hypothesize that these restriction factors may prevent further movement of the transcytosed virions (50). HIV-1 can also pass freely through the intercellular gaps in the vaginal epithelium. We observed a dramatic downregulation of several genes of the plasma membrane and cytoskeleton framework along with downregulation of tight junction proteins (claudins and occludin) induced by the virus. Although the EpiVaginal tissue attempts to mount an interferon response, the excessive inflammation and a disturbance in cellular functions weaken the epithelial barrier and provide a gateway to the underlying target cells (Figure 11A). There are several compelling evidence of interaction of HIV-1 with the vaginal epithelial cells via TLR2 and TLR4 (46), gp340 (51), syndecans (52), and human mannose receptor (53). These receptors, when engaged with PAMPs, initiate an inflammatory cascade. In our model, MALP-2 that activated the TLR2/6 inflammatory axis, synergizes with HIV-1 to further reduce the expression of tight junction proteins and enhances chemokine secretion, reiterating their crucial role in HIV transmission.

The gene signature of EpiVaginal tissues, induced by HIV-1, reflected key mechanisms for viral movement across the multilayered epithelium resulting in its acquisition by the underlying PBMCs. RfhSP-D showed a remarkable ability to restrict viral movement (though not a complete blockade in the experimental conditions). Previously, we and others have shown that rfhSP-D (as well as native SP-D) potently binds to HIV-1 gp120, leading to agglutination and inhibition of infectivity of target cells (19, 54). It can, therefore, be considered that interaction of trimeric rfhSP-D with HIV-1 plausibly results in large complexes that are unable to travel through the tight vaginal barrier. Moreover, since HIV-1 envelope protein gp120 primarily makes the first contact with the epithelium, restriction of this interaction by rfhSP-D may also contribute to a shift from HIV-1 induced gene signature. The presence of a fraction of the virions in the basal chamber of rfhSP-D treated EpiVaginal tissues indicated interaction of the HIV-1 with the EpiVaginal tissues (although it was significantly reduced). In addition, evident from the differential gene expression of the rfhSP-D treated EpiVaginal tissues, rfhSP-D directly interacted with the vaginal epithelial cells and thus strengthened the barrier with upregulated expression of cytoskeleton-related genes. Taken together, these observations suggest that rfhSP-D was able to contain the HIV-1 induced changes in gene expression of the EpiVaginal tissues. Significant upregulation of transcripts for several tight junction proteins in the HIV-1 challenged EpiVaginal tissues in presence of rfhSP-D validated this hypothesis. The two key pro-inflammatory players, NF-κB and mTOR (55, 56), were significantly downregulated, suggesting likely inhibition of the sequential steps of HIV-1 transmission. Notably, the Guanylate binding proteins (GBPs) were either upregulated or unaltered, suggesting that rfhSP-D facilitated the protective response mounted by the EpiVaginal tissue against HIV-1 (Figure 11B). We have recently reported DC-SIGN as a novel receptor of SP-D (using rfhSP-D). A tripartite engagement between DC-SIGN, rfhSP-D and gp120 significantly inhibited transfer of HIV-1 from DC-SIGN to the PBMCs (57). This finding may hold immense importance in vaginal transmission of HIV-1, since DC-SIGN on dendritic cells acts as “Trojan horse” that captures HIV-1 in the mucosa and facilitates its transport to secondary lymphoid organs rich in CD4^+^ T cells followed by trans-infection (58).

SP-D has been shown to potently inhibit the infectivity of other enveloped viruses, such as Influenza A Virus (IAV) (59) and Respiratory Syncytial Virus (RSV) (60), concomitant with induction of an anti-inflammatory environment by interacting with mucosal epithelial and immune cells. This unique property has made rfhSP-D a viable therapeutic option for cystic fibrosis, neonatal lung disease and smoking-induced emphysema (61). RfhSP-D seems to have a similar role against HIV-1 at the vaginal interface. While rfhSP-D limits viral access, it also induces a state of immune quiescence in the vaginal tissues. There is a direct correlation of immune quiescence at the mucosal sites, with resistance to HIV-1 acquisition in serodiscordant women (62). It would be worth exploring the clinical significance of the candidate genes associated with restricted transmission identified in the present study in the highly exposed seronegative women.

An anti-HIV molecule can be effective as a microbicide only if it retains its anti-viral activity without inducing immune activation. Several candidates have failed in the clinical trials due to inflammation caused to the epithelial and target cells, leading to enhanced susceptibility to the virus. Our model revealed that treatment with rfhSP-D did not induce any aberrant inflammatory response by EpiVaginal tissues and did not lead to activation of PBMCs (target cells), and thus, minimized the likelihood of viral transfer and acquisition. In contrast, MALP-2 showed increased activation and susceptibility of PBMCs to the virus, confirming the appropriateness of the model for the evaluation microbicides (63).

To save time and resources, an extensive characterization of candidate prophylactics is warranted before testing their efficacy *in vivo*. Therefore, we subjected rfhSP-D to a series of safety evaluations. RfhSP-D was well-tolerated by human vaginal and ectocervical cells; even at the highest concentration (100 μg/ml), no apparent alterations in the viability of vaginal epithelial cells or inflammation were observed. Similarly, rfhSP-D did not adversely affect the growth of lactobacilli or acid production. However, in the vagina, the epithelial cells and microflora together determine the vaginal health. Vaginal microflora is critical in regulating the epithelial innate immune response. To accurately replicate the *in vivo* condition, we tested safety of rfhSP-D in an epithelial-bacterial colonization model (6). As is the case for a successful microbicide candidate, rfhSP-D did not affect lactobacilli counts, NF-kB activation and chemokine levels in the co-culture. Although SP-D potently inhibits reproductive tract pathogens, such as Chlamydia (64) and Candida (65), we report the SP-D-commensal interaction for the first time. Further investigations may ascertain molecular determinants that define the ability of SP-D to differentiate between vaginal pathogens and commensals. One plausible reason could be evolution of tolerance of the vaginal microflora in the presence of SP-D and other anti-microbial proteins and peptides naturally secreted in the vagina (66). SP-D is naturally expressed and secreted by the human vaginal epithelial cells. Therefore, it was expected that repeated application of rfhSP-D may not harm the vaginal surface. There were no evident histological signs of mucosal toxicity in the rabbit vagina, suggesting that rfhSP-D is well-tolerated *in vivo*.

In summary, we demonstrate the transcriptional gene expression signatures of EpiVaginal tissues in response to HIV-1. An *ex vivo* model of vaginal transmission of HIV-1 was developed that revealed novel genes and features of HIV-1 transmission, and offers a highly reproducible, cost-effective, non-animal model to study efficacy of candidate microbicides. Importantly, rfhSP-D emerges as a potent anti-HIV-1 microbicide candidate, and the results provide a strong argument for its further evaluation in non-human primate models.

## Ethics Statement

Cervicovaginal lavage (CVL) was collected from the normal cycling females with an approval from the Institutional Ethics Committee for Clinical Studies, ICMR-NIRRH (Project No. 148/2008). Blood (*n* = 5) was collected from healthy donors (as determined by clinical examination) at the Department of Pathology, Brigham and Women's Hospital, Boston, MA, under the Partners Healthcare IRB approval (2003P002150). Written informed consent was obtained from each participant. The rabbit vaginal irritation study was approved by the “Institutional Animal Ethics Committee (IAEC),” ICMR-NIRRH, Mumbai (Project No. 08/2012). The IAEC has been recognized by the central organization “Committee for the Purpose of Control & Supervision of Experiments on Animals (CPCSEA).” We strictly adhered to the CPCSEA protocols and guidelines for animal care during the animal experimentation.

## Author Contributions

HP conceived the study, designed, performed and analyzed the experiments, and wrote the paper. KK carried out the RVI model study. KK, GT, and SR carried out the primer designing and Real-time RT-PCR validation of the gene expression. HY conducted the cell viability assessment, recruitment of study participants, and data analysis. PC and MV carried out the microarray analysis, pathway analysis, and presentation. UK provided rfhSP-D for the study and critical suggestions for the manuscript. TM conceived and co-ordinated the study, procured the intra-mural grant and ICMR-Medical Innovation Fund support, mediated the clinical collaboration, defended the protocol for IEC approval, analyzed the data, and edited the paper. RF conceived and co-ordinated the study, facilitated HP's experimentation at BWH, analyzed the data, and edited the paper. All authors reviewed the results and approved the final version of the manuscript.

### Conflict of Interest Statement

The authors declare that the research was conducted in the absence of any commercial or financial relationships that could be construed as a potential conflict of interest. The handling Editor declared a past co-authorship with the authors UK and TM.
