# Supplementary material for: Surfactant Protein D Reverses the Gene Signature of Transepithelial HIV-1 Passage and Restricts the Viral Transfer Across the Vaginal Barrier
Source: Front Immunol. 2019 Mar 28;10:264. doi: 10.3389/fimmu.2019.00264 (PMC6447669; doi:10.3389/fimmu.2019.00264)
Supplement: Supplementary file 1 [file Table_1.docx]

| Gene Name | HIV-1  (FC) | | rfhSP-D + HIV-1 (FC) | | Functions | Ref | Role in HIV | Ref | |
| --- | --- | --- | --- | --- | --- | --- | --- | --- | --- |
| Inflammation | | | | | | | | | |
| ADAM17 | Up  (1.51) | | Down  (-1.16) | | A protease critical in cleavage of TNF-α and other inflammatory proteins to active form. Important in diverse cellular processes such proliferation, migration, cell adhesion | PMID: 20184396 | Nef activates and shuttles activated ADAM17 into exosomes  Exosomal  Nef and ADAM17 activates quiescent CD4+ T Lymphocytes via TNF-α | PMID: 23317503  PMC4178784 | |
| MMP9 | Down  (-3.69) | | Up  (3.75) | | Proteolytic enzyme, degrades extracellular matrix. | PMID: 12540195 | Induced by Tat in astrocytes  Upregulated by gp120 in vaginal epithelial cell line | PMC2679334  PMC3222676 | |
| MYD88 | Up  (1.82) | | Down  (-1.26) | | universal adapter protein downstram of TLRs (except TLR 3) to activate the transcription factor NF-κB | PMID 18064347 | HIV-1 Tat Activates both the MyD88 and TRIF Pathways To Induce TNF-α and IL-10 in Monocytes | PMID: 27053552 | |
| RIPK1 | Up  (1.29) | | Down  (-1.95) | | Serine/threonine kinase that regulate a variety of cellular processes such as cell death and innate immune responses to viral and bacterial infection, induces necroptosis | PMID: 19524512 PMID: 24129419 PMID: 26086143 | Cleaved by HIV proteases and modulate cellular response | PMC4546280 | |
| CD58 | Up  (1.64) | | Down  (-1.25) | | Interaction between CD2 and its counterreceptor, CD58 (LFA-3) aids in T cell-APC cell cell contact | PMID: 10380930 | Engagement of CD58 enhances HIV-1 replication in monocytic cells | PMID: 8656013 | |
| TFF2 | Up  (1.99) | | Down  (-2.11) | | Secreted into the mucus layer where it stabilizes the mucin gel layer and stimulates migration of epithelial cells. Upgregulated in chronic inflammation | PMID: 19064997 | - | - | |
| SERPINE1 | Down  (-2.04) | | Up  (2.92) | | An inhibitor of fibrinolysis, high concentrations of the gene product are associated with thrombophilia | PMID: 24669362 | Monocytes from asymptomatic viremic HIV(+) individuals show increased PAI-1 (SERPINE1) | PMID: 22815948 | |
| CCL20 | Up  (2.94) | | Down  (-2.61) | | Responsible for the chemo-attraction of iDCs, effector/memory B cells and T cells. High specificity for CCR6 | PMID: 27617163 | Attracting key immune cells, including Th17 cells and dendritic cells, to sites of infection and propagating the virus to other sites of the body | PMID: 28005525 | |
| TRIM21 | Up  (3.29) | | Down  (1.08) | | Intracellular antibody effector in the [intracellular antibody-mediated proteolysis](https://en.wikipedia.org/wiki/Intracellular_antibody-mediated_proteolysis) pathway. Directs the virions to the [proteasome](https://en.wikipedia.org/wiki/Proteasome). | PMID 21045130 | Chimeric restriction factor TRIM21-CypA provides highly potent protection against HIV-1 without loss of normal innate immune TRIM activity | PMID: 22909012 | |
| SOCS2 | down  (-1.2) | | Up  (1.63) | | Down-regulation of cytokine signaling | PMID: 12208853 | Tat impaired the IFN γ - receptor signaling pathway at the level of STAT1 activation, via Tat-dependent induction of suppressor of cytokine signaling-2 (SOCS-2) activity | PMID: 19279332 | |
| SOCS3 | Down  (1.09) | | Up  (1.9) | | Down-regulation of cytokine signaling | PMID: 9202125 PMID: 9430658 PMID: 9857039 | Protein levels were lower in CD4 (+) T cells of HIV-infected patients than in healthy controls, Suppressed Th17 levels correlate with elevated SOCS3 expression in CD4 T cells during acute simian immunodeficiency virus infection | PMID: 21337543  PMI21337543D: 23596301 | |
| NOS3 | Up  (1.16) | | Down  (-1.14) | | Major determinant of vascular tone and blood pressure | PMID: 7514568 | Nitric oxide inhibits HIV tat-induced NF-kappaB activation | PMID: 10393859 | |
| PYCARD | Down  (-1.64) | | Up  (2.47) | | Involved in NLRP3 induced inflammasome. Responsible for cleavage of pro-caspase 1 | PMID: 20303873 | Involved caspase-1 dependent pyroptosis of HIV infected CD4 T cells | PMC4047036 | |
| SMARCD1 | Down  (-1.95) | | Up  (2.45) | | Part of SWI/SNF complexes that regulate gene activity of chromatin remodeling, may act as tumor suppressor | PMCID: PMC5406539 | Role in HIV-1 assembly, interaction between Nef and INI1/SMARCB1 augments replicability of HIV-1 in resting PBMCs facilitate Tat-mediated HIV-1 transcription | PMID: 27558426 PMID: 25559666 PMID: 16889668 | |
| CREB1 | Up  (1.69) | | Down  (-2.04) | | CREB family of transcription factors consists of cAMP-responsive activators including CREB, cAMP response element modulator, and activating transcription factor | PMID: 10872467 | Tat utilizes CREB to promote IL-10 production, although the significance of this regarding HIV pathogenesis is not entirely clear, IL-10 can inhibit HIV-1 replication in monocytes and macrophages | PMID: 7527449 | |
| RIPK3 | Down  (-1.28) | | Up  (1.64) | | Serine/threonine kinases that regulate a cellular processes such as cell death and innate immune responses to viral and bacterial infection, induces necroptosis | PMID: 19524512 PMID: 24129419 PMID: 26086143 | Not cleaved by HIV proteases and modulate cellular response | PMC4546280 | |
| SOD1 | Down  (-1.30) | | Up  (1.23) | | Enzyme attaches (binds) to molecules of copper and zinc to break down toxic, charged oxygen molecules called superoxide radicals. | PMID: 7901908 | SOD1 prevented gp120 and Tat elicited reactive oxygen species (ROS) and rescued neuron apoptosis | PMID: 17336361 | |
| TGFBR2 | Up  (1.21) | | Down  (-1.7) | | TGF-β mediates its actions through heteromeric kinase receptor complex consisting of TGF receptors of type 1 and 2 | PMID: 1333888 | Increased expression upon Tat treatment of epithelial cells | PMID: 15857508 | |
| TGFA | Up  (1.15) | | Down  (-1.46) | | Exerts several effects on target cells, such as neovascularization promotion and mitogenic signaling. | PMID: 9242560 | Significant rise in chronic HIV type 1 infection | PMID: 27268396 | |
| SMAD6 | Down  (-1.37) | | Up  (1.59) | | Smad6 inhibits signaling by the TGF-beta superfamily | PMID: 9335505 | Down-regulated after Tat treatment of U937 macrophages | PMID: 16282533 | |
| STX3 | Up  (2.01) | | Down  (-2.04) | | Potentially involved in secretion of IL-6 from dendritic cells following activation of TLRs | PMID: 25674084 | Depletion of STX3 reduced HCMV production | PMID: 25583387 | |
| XRCC2 | Up  (2.33) | | Down  (-2.38) | | DNA repair protein binding to double stranded breaks | PMID: 10227297 | Suppression of retroviral infection by XRCC2 | PMID: 15297876 | |
| **Cytoskeleton and Cell-cell interaction and integrity** | | | | | | | | | |
| GJA1 | Down  (-3.07) | Up  (3.39) | | Involved in intercellular communication (GJIC) between cells to regulate cell death, proliferation, and differentiation. Involved in inflammation | | PMID 25110696 PMID 25560303 | - | | - |
| CD44 | Down  (-2.42) | Up  (5.25) | | Cell-surface glycoprotein involved in cell–cell interactions, cell adhesion and migration | | PMID: 28546458 | Blocking of HIV entry through CD44–hyaluronic acid interactions. | | PMID: 25155464 PMID: 25320329 |
| CAV1 | Down  (-1.89) | Up  (2.53) | | Cav-1 is enriched in caveolae, involved in endocytosis, signal transduction. Role in innate immune defense, and it regulates macrophage cytokine production and signaling | | PMID: 16982844 | Cav-1Tat induced alterations of tight junction protein. Cav-1 mediated uptake via langerin restricts HIV-1 infectivity | | PMID: 18667611, PMID: 25551286 |
| CAV2 | Down  (-1.22) | Up  (2.48) | | Similar to Cav-1 and also inhibits cell proliferation, migration and invasion | | PMID:23454155 | - | | - |
| DBN1 | Down  (-1.86) | Up  (1.74) | | DBN1 suppresses dynamin-mediated endocytosis via interaction with cortactin. DBN1 restricts the entry of viruses into host cells and more broadly to function as a crucial negative regulator of diverse dynamin-dependent endocytic pathways | | PMID: 28416666 | Drebrin is a negative regulator of HIV entry and HIV-mediated cell fusion. Down-regulation of drebrin expression promotes HIV-1 entry, decreases F-actin polymerization, and enhances profilin local accumulation in response to HIV-1. | | PMID: 23926103 |
| NECTIN1 | Down  (1.04) | Up  (2.94) | | Nectin cell adhesion molecule, plays role in organization of adheren junctions and tight junction | | PMID: 28392352 | HIV-Induced Exposure of Nectin-1 Facilitates HSV-1 Infection | | PMID: 24586397 |
| IGFBP3 | Down  (-1.05) | Up  (2.11) | | Binds IGF-I and IGF-II with relatively low affinity, and belongs to a subfamily of low-affinity IGFBPs. It also stimulates prostacyclin production and cell adhesion. | | PMID:21835307 | Inhibit the replication of HIV-1 in cultured cord blood mononuclear cells and chronically HIV-infected U937 cells. | | PMID: 7576911 |
| ACTN1 | Down  (-1.01) | Up  (1.34) | | major actin cross-linking proteins found in virtually all cell types as a cytoskeleton . | | PMID: 26312134 | α-Actinin regulates the immune synapse formation and is required for efficient T cell activation. silencing of either EWI-2 or α-actinin-4 increased cell infectivity. Regulation of the actin cytoskeleton at T cell immune and virological synapses, | | PMID: 22689882 |
| GJB6 | Down  (-1.95) | Up  (2.87) | | Gap junctions allow the transport of ions and metabolites between the cytoplasm of adjacent cells | | PMID: 19944606 | Gap junction channels shutdown under inflammatory conditions, including viral diseases. | | PMCID: PMC4774036 |

**Table 1. rfhSP-D-mediated reversal of HIV-1 induced alteration of gene expressions in EpiVaginal tissues**
