# Supplementary material for: Surfactant Protein D Reverses the Gene Signature of Transepithelial HIV-1 Passage and Restricts the Viral Transfer Across the Vaginal Barrier
Source: Front Immunol. 2019 Mar 28;10:264. doi: 10.3389/fimmu.2019.00264 (PMC6447669; doi:10.3389/fimmu.2019.00264)
Supplement: Supplementary file 2 [file Table_2.DOCX]

**Supplementary Information**

**Surfactant Protein D reverses the gene signature of transepithelial HIV-1 passage and restricts the viral transfer across the vaginal barrier**

Hrishikesh Pandit^1, 2^, Kavita Kale^1^, Hidemi Yamamoto^2^, Gargi Thakur^1^, Sushama Rokade^1^, Payal Chakraborty^3^, Madhavan Vasudevan^3^, Uday Kishore^4^, Taruna Madan^1,*^, Raina Fichorova^2, *^

^1^ICMR-National Institute for Research in Reproductive Health, Innate Immunity, Mumbai, India,

^2^Harvard Medical School and Brigham and Women's Hospital, Laboratory of Genital Tract Biology, Boston, MA, United States

^3^Genome Informatics Research Group, Bionivid Technology Pvt. Ltd., Bengaluru, India

^4^Biosciences, College of Health and Life Sciences, Brunel University London, United Kingdom

*Correspondence:

Taruna Madan

[taruna_m@hotmail.com](mailto:taruna_m@hotmail.com)

Raina Fichorova

[rfichorova@rics.bwh.harvard.edu](mailto:rfichorova@rics.bwh.harvard.edu)

Running Title: SP-D restricts transepithelial HIV-1 passage

1. **Supplementary Figure Legends**

**1.1 Supplementary Fig. S1 Microarray data of EpiVaginal tissues treated with HIV-1 Vs untreated: (A)** Hierarchical clustering of differentially expressed genes and **(B)** Scatter plot identifying significantly differentially expressed transcripts. Of the total 355 differentially regulated genes, 187 were upregulated and 168 were downregulated.

**1.2 Supplementary Fig. S2 Microarray data of EpiVaginal tissues treated with rfhSP-D + HIV-1 Vs HIV-1: (A)** Hierarchical clustering of differentially expressed genes and **(B)** Scatter plot identifying significantly differentially expressed transcripts. Of the total 518 differentially regulated genes, 363 were upregulated and 155 were downregulated.

**1.3 Supplementary Fig. S3** Complete regulatory gene network of rfhSP-D + HIV-1 Vs HIV-1 challenged EpiVaginal tissues. Three biological processes are blue colored blocks and downregulated genes are green colored, upregulated are red, unaltered are orange. Circles are sized according to their p Value.

**1.4 Supplementary Fig. S4 Microarray data of EpiVaginal tissues treated with rfhSP-D Vs Untreated: (A)** Hierarchical clustering of differentially expressed genes and **(B)** Scatter plot identifying significantly differentially expressed transcripts. Of the total 185 differentially regulated genes, 103 were upregulated and 82 were downregulated.

- 1. **Supplementary Fig. S5 Expression of SP-D in the vaginal milieu. (A)** Western blot analysis of human vaginal lavage probed with monoclonal antibody to human SP-D: lane 1 - Rainbow ladder, lane 2 - amniotic fluid (positive control), lane 3 - proliferative phase vaginal lavage, lane 4 - negative control. A specific band at 43 kDa in lane 2 and 3 confirmed presence of SP-D. **(B)** RT-PCR of vaginal epithelial (Vk2/E6E7) cells showing specific transcripts of SP-D (143 bp) and non-template controls (NTC). Lane 1 is a 100 bp ladder. **(C)** Confocal microscopy of Vk2/E6E7 cells showing cytoplasmic presence of SP-D (upper panel) and negative control (Lower panel). Magnification: 40X
