# Supplementary figures and images for: Surfactant Protein D Reverses the Gene Signature of Transepithelial HIV-1 Passage and Restricts the Viral Transfer Across the Vaginal Barrier
Source: Front Immunol. 2019 Mar 28;10:264. doi: 10.3389/fimmu.2019.00264 (PMC6447669; doi:10.3389/fimmu.2019.00264)

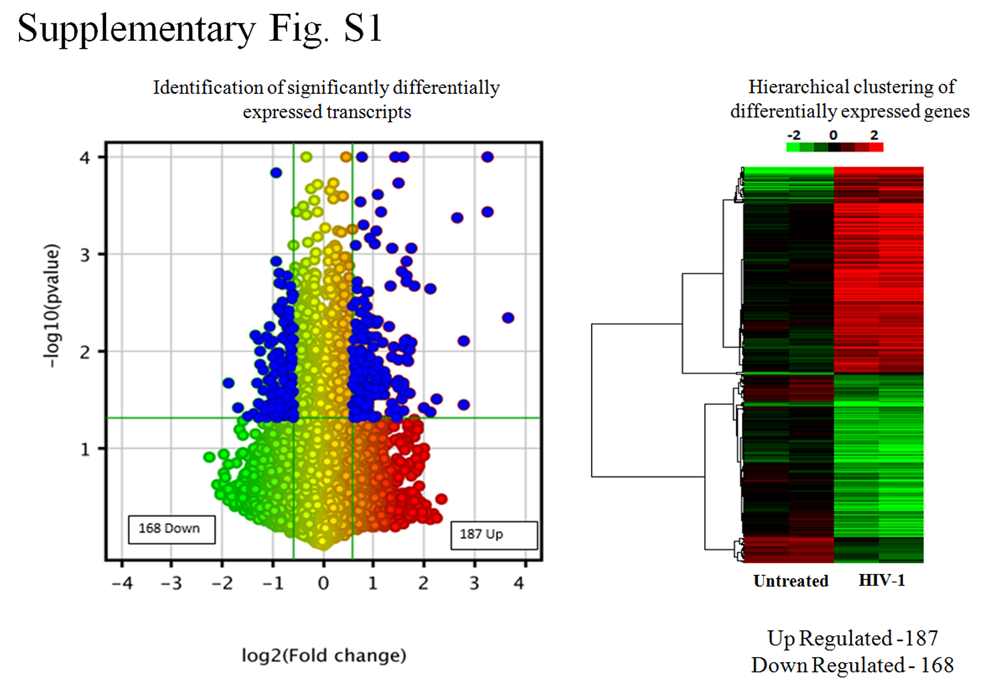

Supplement: Supplementary file 3 [file Image_1.TIF]

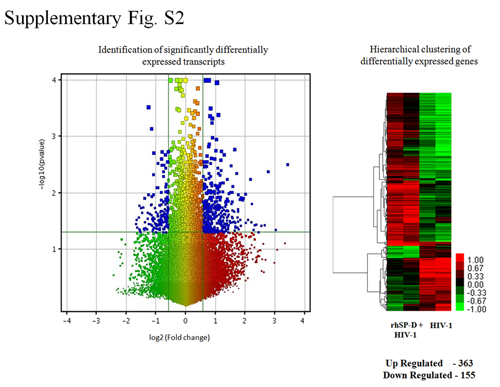

Supplement: Supplementary file 4 [file Image_2.TIF]

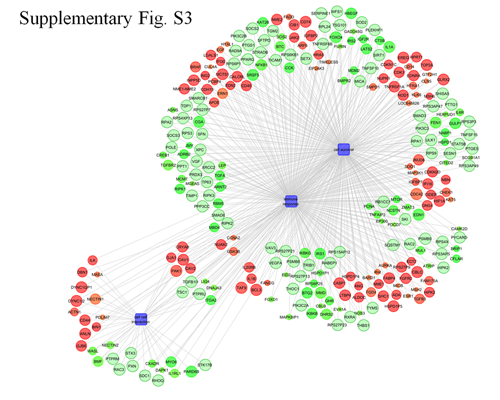

Supplement: Supplementary file 5 [file Image_3.TIF]

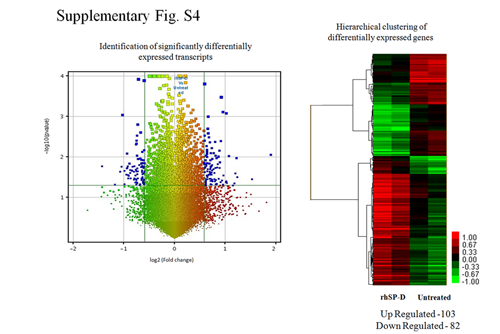

Supplement: Supplementary file 6 [file Image_4.TIF]

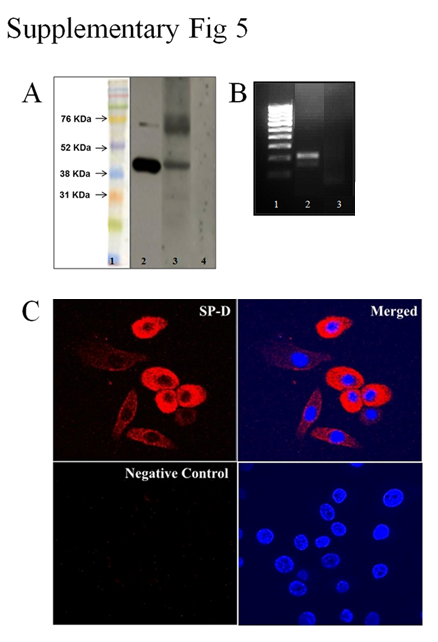

Supplement: Supplementary file 7 [file Image_5.TIF]
